# Supplementary material for: In Silico Exploration of the Trypanothione Reductase (TryR) of L. mexicana
Source: Int J Mol Sci. 2023 Nov 7;24(22):16046. doi: 10.3390/ijms242216046 (PMC10671491; doi:10.3390/ijms242216046)
Supplement: Supplementary file 1 [file ijms-24-16046-s001.zip › ijms-2589979-supplementary.pdf]

In silico exploration of the trypanothione reductase (TryR) of *L. mexicana*

Francisco J. Barrera-Téllez[1], Fernando D. Prieto-Martínez[2], Alicia Hernandez-Campos[1], Karina Martínez-Mayorga[3], Rafael Castillo-Bocanegra*[1]

[1] *Departamento de Farmacia, Facultad de Química, Universidad Nacional Autónoma de México, CP 04510, Ciudad de México, México; rafaelc@unam.mx, hercam@unam.mx, frank.ben.alfred@gmail.com*

[2] *Instituto de Química, Unidad Mérida, Universidad Nacional Autónoma de México, Carretera Mérida-Tetiz, Km. 4.5, Ucú, Yucatán, México; ferdpm4@hotmail.com*

[3] *Instituto de Investigaciones en Matemáticas Aplicadas y en Sistemas, Unidad Mérida, Universidad Nacional Autónoma de México, Sierra Papacal, Mérida, Yucatán, México; kmtzm@unam.mx*

Table of contents

Table S1. Comparison between the reported crystal structures of TryR from *L. infantum* and the homology models with the SwissModel and SAVES servers.

Table S2. Masked residues in 2W0H and TRLMS for FTMap server.

Table S3. Nonbonded interactions (noncovalent contacts) for 2W0H and TRLMS.

Table S4. Hydrogen bond interactions for 2W0H and TRLMS.

Table S5. CSs located by FTMap in 2W0H and TRLMS.

Table S6. CryptoScore of selected residues in 2W0H and TRLMS.

Table S7. Composition of cavities found by DogSite3 in 2W0H.

Table S8. Composition of cavities found by DogSite3 in TRLMS.

Table S9. Composition of cavities found by Desmond in TRLMS.

Table S10. Composition of cavities found by Desmond in TRLMS and 2W0H.

Table S11. Comparison of key residues in the TryR of various parasites.

Table S12. Comparison of key residues between TryR and GR (glutathione reductase).

Figure S1. Comparative of Root-mean squared distance (RMSD), Root-mean squared fluctuation (RMSF) and Radius of gyration of the two chains of TryR in simulated systems.

Figure S2. Ramachandran number histograms, as obtained with Backmap for the simulated systems.

Figure S3. Secondary structure distribution, as obtained with Backmap for the simulated systems.

Figure S4. Secondary structure deviation, as obtained with Backmap for the simulated systems.

Figure S5. Secondary structure fluctuation, as obtained with Backmap for the simulated systems.

Figure S6. Residues marked as hot spots by FHM in 2W0H.

Figure S7. Residues marked as hot spots by FHM in TRLMS.

Figure S8. Residues marked as cryptic sites by CryptoSite in 2W0H.

Figure S9. Residues marked as cryptic sites by CryptoSite in TRLMS.

Figure S10. Most representative cavities found by DogSite3 in 2W0H.

Figure S11. Most representative cavities found by DogSite3 in TRLMS.

Figure S12. Most representative cavities found by Desmond in TRLMS (5 ns, Experiment A).

Figure S13. Most representative cavities found by Desmond in TRLMS (20 and 40 ns, Experiment A).

Figure S14. Most representative cavities found by Desmond in 2W0H (20 ns, Experiment B).

Figure S15. Most representative **c**avities found by Desmond in TRLMS (20 ns, Experiment C).

Figure S16. Free energy surfaces obtained from six independent runs of metadynamics simulations of 2W0H.

Figure S17. Free energy surfaces obtained from duplicate runs of metadynamics simulations of TRLMS.

Figure S18. Radius of gyration of the residues that integrate the σ-site as recovered from the initial simulations of homology models of TRLMA, TRLMS and 2W0H.

Figure S19. Histograms of radius of gyration values obtained from classical molecular dynamics simulations of TRLMS and 2W0H.

Figure S20. Histograms of radius of gyration values obtained from metadynamics simulations of TRLMS and 2W0H.

Figure S21. Sequence comparison between GR (3DJJ), TRLMS and 2JK6.

Figure S22. Free energy surfaces obtained from six independent runs of metadynamics simulations in GR for σ-site.

Figure S23. Native conformation of compound 71 in PDBID 5S9W.

Figure S24. Overlay of TRLMS σ-site with the PDBIDs 5S9W and 3DJJ.

Figure S25. Free energy surface for PLANTS´ top pose of compound 71 in TRLMS (independent replica).

Figure S26. Free energy surface for LeDock´s top pose of compound 71 in TRLMS (independent replica).

Figure S27. Free energy surface for PLANTS' top pose of compound 71 in GR (independent replica).

Figure S28. Free energy surface for LeDock's top pose of compound 71 in GR (independent replica).

Figure S29. Free energy surface for PLANTS' top pose of ZINC12151998 in TRLMS (independent replica).

Figure S30. Free energy surface for LeDock's top pose of ZINC12151998 in TRLMS (independent replica).

Figure S31. Free energy surface for PLANTS' top pose of ZINC12151998 in GR (independent replica).

Figure S32. Free energy surface for LeDock's top pose of ZINC12151998 in GR (independent replica).

Table S1. Comparison between the reported crystal structures of TryR from *L. infantum* and the homology models with the SwissModel and SAVES servers.

Server or portal	Code	RMSD[1]	QMEAN4[2]	QMEANDisCo[2]	Ramachandran plot (F/A)[3]
RCSB	2JK6	n/a[4]	-0.52	0.87 ± 0.05	89.7/9.5
RCSB	2WOH	n/a[4]	-0.48	0.87 ± 0.05	87.3/12.1
Swiss Model	TRLMS	0.606[5]/0.675[6]	-0.90	0.83 ± 0.05	86.5/12.5
AlphaFold	TRLMA	0.624[5]/0.538[6]	-0.42	0.85 ± 0.05	89.8/9.8

[1]RMSD = Root-mean-square deviation; [2]QMEAN = Qualitative Model Energy Analysis; [3]Ramachandran plot regions: F (residues in most favored regions) and A (residues in additional allowed regions), these data were collected from the PROCHECK server that is included in SAVES; [4]n/a = not applicable; [5]RMSD calculated with respect to PDBID 2JK6; [6]RMSD calculated with respect to PDBID 2W0H.

Table S2. Masked residues in 2W0H and TRLMS for FTMap server.

Sequences[1]	Residues[2]
2W0H (*L. infantum*)	<u>Leu10</u>, Gly11, Ala12, Gly13, Gly15, Gly16, Val34, Asp35, <u>Val36</u>, Ala46, Ala47, Gly50, Thr51, Val55, Gly56, Lys60, Gly125, Phe126, Gly127, Ala159, Thr160, Gly161, Ser162, Phe182, Val194, Gly195, Gly196, Gly197, Ile199, Ala200, Glu202, Phe203, Tyr221, Arg222, Arg228, <u>Asn254</u>, Ala284, Ile285, Gly286, Arg287, Arg290, Leu294, Gly326, Asp327, Met333, Leu334, Ala338, Val366, Phe367, Val460
TRLMS (*L. mexicana*)	<u>Ile10</u>, Gly11, Ala12, Gly13, Gly15, Gly16, Val34, Asp35, <u>Leu36</u>, Ala46, Ala47, Gly50, Thr51, Val55, Gly56, Lys60, Gly125, Phe126, Gly127, Ala159, Thr160, Gly161, Ser162, Phe182, Val194, Gly195, Gly196, Gly197, Ile199, Ala200, Glu202, Phe203, Tyr221, Arg222, Arg228, <u>Ser254</u>, Ala284, Ile285, Gly286, Arg287, Arg290, Leu294, Gly326, Asp327, Met333, Leu334, Ala338, Val366, Phe367, Val460

[1]The residues indicated in each sequence were masked in both chains; [2]Residues that differ between sequences are underlined.

Table S3. Nonbonded interactions (noncovalent contacts) for 2W0H and TRLMS.[1]

	2W0H (Chain A)		TRLMS (Chain A)		2W0H (Chain B)		TRLMS (Chain B)	
Zone/ Pocket[2]	Residue[3]	N. C.[4]	Residue	N. C.	Residue	N. C.	Residue	N. C.
C.C.	Gly13	613	Gly13	3232	Gly13	453		
C.C.	Ser14	2338	Ser14	6639	Ser14	1934		
C.C.	Gly49	717	Gly49	1235	Gly49	570		
C.C.	Thr51	1974	Thr51	872	Thr51	2091		
C.C.	Val53	445	Val53	512	Val53	506		
C.C.	Gly56	1816	Gly56	1760	Gly56	1215		
C.C.	Lys61 (σ)	13404	Lys61 (σ)	20350	Lys61 (σ)	11955	Lys61 (σ)	17495
C.C.	Pro336 (λ)	1811	Pro336 (λ)	3535	Pro336 (λ)	1074	Pro336 (λ)	3925
C.C.							Asn340 (λ)	485
C.C.			Glu410	1826			Glu410	531
C.C.			Thr457	868				
C.C.			Ile458 (λ)	2069				

C.C.			Gly459 (λ)	838				
C.C.	Val460	960	Val460	4552				
C.C.	Pro462 (σ)	6631	Pro462 (σ)	8346	Pro462 (σ)	7778	Pro462 (σ)	5169
C.C.	Thr463 (σ)	8000	Thr463 (σ)	12060	Thr463 (σ)	9115	Thr463 (σ)	1520
C.C.	Ser464 (σ)	8280	Ser464 (σ)	12769	Ser464 (σ)	7718	Ser464 (σ)	4117
C.S.	Cys52	2796	Cys52	1969	Cys52	2801	Cys52	457
C.S.	Cys57	8339	Cys57	12511	Cys57	5390	Cys57	2426
C.S./γ-g.s.	His461 (λ)	6432	His461 (λ)	18415	His461 (λ)	9979	His461 (λ)	6305
γ-g.s.					Glu466 (λ)	593		
H.C.			Leu17	1367				
H.C.	Tyr110	1116			Tyr110	611		
H.C.	Thr335	10249	Thr335	7187	Thr335	7738	Thr335	7887
H.C.			Ile339 (λ)	415				
Z-site	Phe396 (σ)	2283			Phe396 (σ)	4435		
Z-site	Pro398 (σ)	6263	Pro398 (σ)	3438	Pro398 (σ)	5419	Pro398 (σ)	1968
Z-site	Leu399 (σ)	2689			Leu399 (σ)	2805		
N.S.	Tyr198 (Δ)	672	Tyr198 (Δ)	2739			Tyr198 (Δ)	1283
N.S.	Ile199	714	Ile199	574			Ile199	461
N.S.	Glu202	1368	Glu202	1497	Glu202	621	Glu202	1576
N.S.	Met333 (Δ)	1705	Met333 (Δ)	2291	Met333 (Δ)	1536	Met333 (Δ)	1486
N.S.	Leu334 (Δ)	11383	Leu334 (Δ)	13385	Leu334 (Δ)	7266	Leu334 (Δ)	12438
N.S.	Cys364 (Δ)	494	Cys364 (Δ)	703			Cys364 (Δ)	1117
N.S.	Ala365 (Δ)	1847	Ala365 (Δ)	2248	Ala365 (Δ)	910	Ala365 (Δ)	2327
Interface	Lys60	3340	Lys60	12059	Lys60	1649	Lys60	2957
Interface	Val64	588	Val64	13875	Val64	483	Val64	9156
Interface	Thr65	40380	Thr65	25132	Thr65	47063	Thr65	21283
Interface	Gln68	57651	Gln68	38071	Gln68	49046	Gln68	47563
Interface	Tyr69	26651	Tyr69	18061	Tyr69	34336	Tyr69	17487
Interface	Asp71	1620						
Interface	Leu72	30383	Thr72	17776	Leu72	18281	Thr72	4575
Interface	Glu75	2800	Glu75	7216	Glu75	1249		
Interface	Phe367	10701	Phe367	28933	Phe367	8036	Phe367	24823
Interface			Ser368	1390			Ser368	771
Interface	Pro371	10997	Pro371	7786	Pro371	9140	Pro371	14076
Interface	Met400	13306	Met400	6270	Met400	6719	Met400	1274
Interface	His401	1074	His401	3190	His401	626	His401	1967

Interface	Lys409	1633	Lys409	1174			Lys409	1044
Interface	Phe411	1595	Phe411	4655	Phe411	740	Phe411	4940
Interface	Gly431	600	Gly431	1240			Gly431	1849
Interface	Asp432	23112	Glu432	15839	Asp432	18302	Glu432	30644
Interface	Ser433	24622	Ser433	8245	Ser433	18386	Ser433	23561
Interface	Ala434	743	Ala434	545	Ala434	571	Ala434	2745
Interface	Pro435	12163	Pro435	8426	Pro435	8756	Pro435	30012
Interface	Glu436	18417	Glu436	3807	Glu436	12174	Glu436	17115
F.S.	Asp327	1317	Asp327	1042	Asp327	1417	Asp327	659

[1]Residues marked with yellow were masked in FTMap and were not taken into account; [2]C.C. = Catalytic Cavity, C.S. = Catalytic Site, γ-g.s. = γ-glutamic site, H.C. = Hydrophobic Cleft, N.S. = NADPH Site, F.S. = FAD Site; [3]σ, λ, Δ = sigma, lambda and doorstop sites; [4]N.C. = Number of contacts.

Table S4. Hydrogen bond interactions for 2W0H and TRLMS.[1]

	**2W0H (Chain A)**		**TRLMS (Chain A)**		**2W0H (Chain B)**		**TRLMS (Chain B)**	
**Zone[2]**	**Residue[3]**	**N. C.[4]**	**Residue**	**N. C.**	**Residue**	**N. C.**	**Residue**	**N. C.**
C.C.			Ser14	84				
C.C.					Thr51	10		
C.C.	Lys61 (σ)	563	Lys61 (σ)	182	Lys61 (σ)	611	Lys61 (σ)	755
C.C.			Glu410	38				
C.C.			Pro462 (σ)	55			Pro462 (σ)	94
C.C.	Thr463 (σ)	116			Thr463 (σ)	300		
C.C.	Ser464 (σ)	174	Ser464 (σ)	993	Ser464 (σ)	168	Ser464 (σ)	271
C.S.			Cys52	209				
C.S.	Cys57	74	Cys57	125	Cys57	62	Cys57	70
C.S./γ-g.s.			His461 (λ)	280	His461 (λ)	28	His461 (λ)	185
γ-g.s	Glu466 (λ)	61			Glu466 (λ)	155		
H.C.	Thr335	290	Thr335	421	Thr335	151	Thr335	530
Z-site	Leu399 (σ)	43			Leu399 (σ)	70		
N.S.			Tyr198 (Δ)	14				
N.S.			Glu202	95			Glu202	51
N.S.			Met333 (Δ)	30			Met333 (Δ)	111
N.S.			Ala365 (Δ)	64			Ala365 (Δ)	100
Interface	Lys60	389	Lys60	930	Lys60	164	Lys60	468
Interface	Thr65	383	Thr65	765	Thr65	659	Thr65	772
Interface	Gln68	1228	Gln68	581	Gln68	851	Gln68	1056
Interface	Tyr69	396	Tyr69	83	Tyr69	738	Tyr69	662

Interface			Thr72	465			Thr72	128
Interface	Glu75	147	Glu75	308	Glu75	63		
Interface	Ser368	23	Ser368	49				
Interface	Met400	20						
Interface	His401	37	His401	38	His401	13		
Interface	Lys409	31						
Interface	Asp432	420	Glu432	149	Asp432	426	Glu432	376
Interface	Ser433	1003	Ser433	505	Ser433	1082	Ser433	1482
Interface	Glu436	225	Glu436	84	Glu436	167	Glu436	399
F.S.			Asp327	69	Asp327	15	Asp327	38

[1]Residues marked with yellow were masked in FTMap and were not taken into account; [2]C.C. = Catalytic Cavity, C.S. = Catalytic Site, γ-g.s. = γ-glutamic site, H.C. = Hydrophobic Cleft, N.S. = NADPH Site, F.S. = FAD Site; [3]σ, λ, Δ = sigma, lambda and doorstop sites; [4]N.C. = Number of contacts.

Table S5. CSs located by FTMap in 2W0H and TRLMS.[1]

Code	Number of clusters	Druggable CSs (Number of probes)	Semi-druggable CSs (Number of probes)	No druggable CSs (Number of probes)
2W0H	15	None	CS00(13)	CS01(9), CS02(8), CS03(8), CS04(8), CS05(8), CS06(8), CS07(7), CS08(7), CS09(7), CS10(4), CS11(2), CS12(2), CS13(1), CS14(1)
TRLMS	15	CS00(18)	None	CS01(12), CS02(12), CS03(9), CS04(7), CS05(7), CS06(5), CS07(4), CS08(4), CS09(3), CS10(3), CS11(3), CS12(3), CS13(2), CS14(2)

[1]FTMap establishes that if a CS has more than 16 probes (S > 16) it can be druggable by a ligand and is considered a strong hotspot; consensus clusters of S < 13 occupy sites that are not druggable due to very weak hotspots and between 16 and 13 there is some uncertainty.

Table S6. CryptoScore of selected residues in 2W0H and TRLMS.[1]

	2W0H (Chain A)		TRLMS (Chain A)		2W0H (Chain B)		TRLMS (Chain B)	
Zone/ Pocket[2]	Residue[3]	CrypS[4]	Residue	CrypS	Residue	CrypS	Residue	CrypS
C.C.	Ser14	28.19	Ser14	31.43	Ser14	32.17	Ser14	24.50
C.C.			Val53	10.70	Val53	11.99		
C.C.			Val58	10.43	Val58	10.64		
C.C.	Lys61 (σ)	25.83	Lys61 (σ)	42.35	Lys61 (σ)	22.61	Lys61 (σ)	19.77
C.C.	Pro336 (λ)	10.66	Pro336 (λ)	11.58	Pro336 (λ)	16.01	Pro336 (λ)	20.41
C.C.					His455	11.71		
C.C.	Pro462 (σ)	25.28	Pro462 (σ)	25.31	Pro462 (σ)	18.23	Pro462 (σ)	20,96

C.C.	Thr463 (σ)	25.91	Thr463 (σ)	27.64	Thr463 (σ)	25.44	Thr463 (σ)	25,94
C.C.	Ser464 (σ)	14.77	Ser464 (σ)	14.76	Ser464 (σ)	15.19	Ser464 (σ)	19.54
C.S.	Cys52	18.18	Cys52	21.19	Cys52	22.34	Cys52	18.61
C.S.	Cys57	39.34	Cys57	42.94	Cys57	43.59	Cys57	37.47
C.S./γ-g.s.	His461 (λ)	33.11	His461 (λ)	26.34	His461 (λ)	40.12	His461 (λ)	32.21
γ-g.s.	Glu466 (λ)	29.61	Glu466 (λ)	26.79	Glu466 (λ)	33.12	Glu466 (λ)	33.29
γ-g.s.	Glu467	11.73			Glu467	10.43	Glu467	13.36
H.C.	Tyr110	29.27	Tyr110	23.32	Tyr110	27.69	Tyr110	28.98
H.C.	Thr335	34.92	Thr335	36.10	Thr335	38.31	Thr335	36.26
H.C.	Ile339 (λ)	11.00	Ile339 (λ)	10.67	Ile339 (λ)	12.38	Ile339 (λ)	10.20
Z-site	Phe396 (σ)	25.46	Phe396 (σ)	33.67	Phe396 (σ)	28.35	Phe396 (σ)	35.35
Z-site	Pro398 (σ)	20.74	Pro398 (σ)	14.14	Pro398 (σ)	16.47	Pro398 (σ)	14,27
Z-site	Leu399 (σ)	17.23	Leu399 (σ)	17.81	Leu399 (σ)	13.78	Leu399 (σ)	13,09
Y-site	Cys469	14.14	Cys469	11.67	Cys469	18.58		
Y-site	Ser470 (λ)	16.64	Ser470 (λ)	19.09	Ser 470 (λ)	17.40	Ser470 (λ)	12.97
Y-site			Arg472 (λ)	15.05			Arg472 (λ)	10.50
18-g.s.	Glu18	15.22	Glu18	14.32	Glu18	13.56	Glu18	16.41
N.S.							Ser178	11.20
N.S.	Gly229 (Δ)	22.84	Gly229 (Δ)	25.01	Gly229 (Δ)	22.07	Gly229 (Δ)	23.72
N.S.	Phe230 (Δ)	11.96	Phe230 (Δ)	25.04	Phe230 (Δ)	14.61	Phe230 (Δ)	15.42
N.S.			Val362	10.34	Val362	13,01	Val362	13.89
N.S.					Thr374 (Δ)	10.03		
N.S.	Gly376 (Δ)	22.52	Gly376 (Δ)	29.14	Gly376 (Δ)	25.16	Gly376 (Δ)	25.13
Interface					Leu62	10.82		
Interface			Val64	10.68			Val64	11,13
Interface	Thr65	10.77	Thr65	23.39	Thr65	12.42	Thr65	22.86
Interface			Tyr69	17.56				
Interface	Met400	18.46	Met400	16.82	Met400	18.05	Met400	17.94
Interface			His401	10.06			His401	14,57
Interface	Ser433	14.92	Ser433	13.56	Ser433	15.93	Ser433	11,50
Interface			Pro435	10.11				
Interface	Glu436	34.95	Glu436	40.17	Glu436	34.56	Glu436	38.42
N.D.							Lys211	10.54

[1]Only residues with a CryptoScore above 10 were taken into account, according to the recommendations of the CryptoSite portal; [2]C.C. = Catalytic Cavity, C.S. = Catalytic Site, γ-g.s. = γ-glutamic site, H.C. = Hydrophobic Cleft, 18-g.s. = 18-glutamic site, N.S. = NADPH Site, N.D. = Not determined; [3]σ, λ, Δ = sigma, lambda and doorstop sites; [4]CrypS = Crypto Score.

Table S7. Composition of cavities found by DogSite3 in 2W0H.

Cavity	Residues[1,2]	Properties[3]	
1	Chain A = Phe396 (σ), Pro398 (σ), Leu399 (σ), Met400, Asp432, Ser433, Pro462 (σ), Thr463 (σ), Ser464 (σ), Glu466 (λ)<br><br>Chain B = Val58, Lys61 (σ), Leu62, Gln68, Phe367, Pro371, Asp432, Ser433, Pro435, Glu436	V (Å^3) = 277.504<br>S (Å^2) = 456.41<br>LS (Å^2) = 277.488<br>D (Å) = 14.9666<br>Ac = 9<br>Do = 10<br>Ar = 6	H = 0.764706<br>AR = 4<br>BR = 1<br>PR = 5<br>APR = 10<br>TR = 20
2	Chain A = Val58, Lys61 (σ), Leu62, Gln68, Phe367, Pro371, Asp432, Ser433, Pro435, Glu436<br><br>Chain B = Phe396 (σ), Pro398 (σ), Leu399 (σ), Met400, Asp432, Ser433, Pro462 (σ), Thr463 (σ), Ser464 (σ), Glu466 (λ)	V (Å^3) = 265.728<br>S (Å^2) = 468.791<br>LS (Å^2) = 275.847<br>D (Å) = 15.242<br>Ac = 10<br>Do = 9<br>Ar = 5	H = 0.756098<br>AR = 4<br>BR = 1<br>PR = 5<br>APR = 10<br>TR = 20
3	Chain A = His455, Thr457, Ile458 (λ), Gly459 (λ), Glu466 (λ), Cys469, Ser470 (λ), Arg472 (λ)<br><br>Chain B = Glu18, Pro336 (λ), Ile339 (λ), Asn340 (λ), Ala343 (λ), Arg355	V (Å^3) = 159.232<br>S (Å^2) = 355.284<br>LS (Å^2) = 179.094<br>D (Å) = 9.08625<br>Ac = 9<br>Do = 7<br>Ar = 0	H = 0.660377<br>AR = 2<br>BR = 3<br>PR = 5<br>APR = 4<br>TR = 14
4	Chain A = Glu18, Pro336 (λ), Ile339 (λ), Asn340 (λ), Ala343 (λ), Arg355<br><br>Chain B = His455, Thr457, Ile458 (λ), Gly459 (λ), Glu466 (λ), Cys469, Ser470 (λ), Arg472 (λ)	V (Å^3) = 142.848<br>S (Å^2) = 360.849<br>LS (Å^2) = 180.243<br>D (Å) = 8.2365<br>Ac = 8<br>Do = 7<br>Ar = 0	H = 0.659574<br>AR = 2<br>BR = 3<br>PR = 5<br>APR = 4<br>TR = 14
5	Chain A = Trp81<br><br>Chain B = Met70, Ile73, Arg74, Arg85, Leu88	V (Å^3) = 62.464<br>S (Å^2) = 150.5<br>LS (Å^2) = 104.462<br>D (Å) = 5.82409<br>Ac = 3<br>Do = 0<br>Ar = 3	H = 0.83871<br>AR = 0<br>BR = 2<br>PR = 0<br>APR = 4<br>TR = 6
6	Chain A = Met70, Ile73, Arg74, Arg85, Leu88<br><br>Chain B = Trp81	V (Å^3) = 55.808<br>S (Å^2) = 145.828<br>LS (Å^2) = 101.774<br>D (Å) = 5.65685<br>Ac = 3<br>Do = 0<br>Ar = 3	H = 0.857143<br>AR = 0<br>BR = 2<br>PR = 0<br>APR = 4<br>TR = 6
7	Chain A = None<br><br>Chain B = Gln241, Pro370, Pro398 (σ), His401, Lys409, Glu410, Phe411, Leu430, Gly431, Asp432	V (Å^3) = 39.424<br>S (Å^2) = 118.002<br>LS (Å^2) = 67.5791<br>D (Å) = 5.05964<br>Ac = 5<br>Do = 1<br>Ar = 6	H = 0.675676<br>AR = 2<br>BR = 2<br>PR = 2<br>APR = 4<br>TR = 10
8	Chain A = Ser456, Thr457, Ile458 (λ)<br><br>Chain B = Asp358, Thr360 (Δ), Lys361, Val362, Ala363, Gly442, Lys446	V (Å^3) = 38.4<br>S (Å^2) = 160.793<br>LS (Å^2) = 87.057<br>D (Å) = 4.38178<br>Ac = 6<br>Do = 5<br>Ar = 0	H = 0.674419<br>AR = 1<br>BR = 2<br>PR = 4<br>APR = 3<br>TR = 10
9	Chain A = Asp358, Thr360 (Δ), Lys361, Val362, Ala363, Gly442, Lys446	V (Å^3) = 36.352<br>S (Å^2) = 148.518<br>LS (Å^2) = 88.8489	H = 0.697614<br>AR = 1<br>BR = 2

	Chain B = Ser456, Thr457, Ile458 (λ)	D (Å) = 4.38178<br>Ac = 6<br>Do = 4<br>Ar = 0	PR = 4<br>APR = 3<br>TR = 10
**10**	Chain A = Pro370, Pro398 (σ), His401, Lys409, Glu410, Phe411, Gly431, Asp432, Ser464 (σ)<br><br>Chain B = None	V (Å^3) = 34.816<br>S (Å^2) = 136.397<br>LS (Å^2) = 73.4923<br>D (Å) = 4.38178<br>Ac = 7<br>Do = 2<br>Ar = 6	H = 0.694444<br>AR = 2<br>BR = 2<br>PR = 2<br>APR = 3<br>TR = 9

[1]Residues marked with yellow were not taken into account; [2]σ, λ, Δ = sigma, lambda and doorstop sites [3]V = Volume; S = Surface; LS = LipoSurface; D = Depth; Ac = Number of solvent accessible hydrogen bond acceptors; Do = Number of solvent accessible hydrogen bond donors; Ar = Number of all aromatic atoms; H = Hydrophobicity of pocket; AR = Acidic residues; BR = Basic residues; PR = Polar residues; APR = Apolar residues; TR = Total residues.

Table S8. Composition of cavities found by DogSite3 in TRLMS.

**Cavity**	**Residues[1,2]**	**Properties[3]**	
**1**	Chain A = Lys61 (σ), Val64, Thr65, Gln68, Phe367, Pro371, Ser433, Pro435, Glu436<br><br>Chain B = Pro462 (σ)	V (Å^3) = 138.24<br>S (Å^2) = 221.619<br>LS (Å^2) = 112.194<br>D (Å) = 8.0796<br>Ac = 5<br>Do = 5<br>Ar = 4	H = 0.756757<br>AR = 1<br>BR = 1<br>PR = 3<br>APR = 5<br>TR = 10
**2**	Chain A = Pro462 (σ), Thr463(σ)<br><br>Chain B = Lys61 (σ), Val64, Thr65, Gln68, Phe367, Pro371, Glu432, Ser433, Pro435, Glu436	V (Å^3) = 123.392<br>S (Å^2) = 241.023<br>LS (Å^2) = 119.225<br>D (Å) = 7.63151<br>Ac = 5<br>Do = 4<br>Ar = 3	H = 0.761905<br>AR = 2<br>BR = 1<br>PR = 4<br>APR = 5<br>TR = 12
**3**	Chain A = His455, Thr457, Ile458 (λ), Gly459 (λ), Val460, Glu466 (λ), Cys469, Ser470 (λ), Arg472 (λ)<br><br>Chain B = Pro336 (λ), Ile339 (λ), Asn340 (λ), Gln439	V (Å^3) = 120.32<br>S (Å^2) = 264.079<br>LS (Å^2) = 143.216<br>D (Å) = 7.63151<br>Ac = 6<br>Do = 4<br>Ar = 0	H = 0.66<br>AR = 1<br>BR = 2<br>PR = 6<br>APR = 4<br>TR = 13
**4**	Chain A = Pro336 (λ), Ile339 (λ), Asn340 (λ), Ala343 (λ), Arg355<br><br>Chain B = His455, Asn456, Thr457, Ile458 (λ), Gly459 (λ), Glu466 (λ), Cys469, Ser470 (λ), Arg472 (λ)	V (Å^3) = 109.568<br>S (Å^2) = 276.52<br>LS (Å^2) = 102.227<br>D (Å) = 8.19756<br>Ac = 9<br>Do = 7<br>Ar = 0	H = 0.586957<br>AR = 1<br>BR = 3<br>PR = 6<br>APR = 4<br>TR = 14
**5**	Chain A = Lys61 (σ)<br><br>Chain B = Gln241, Pro370, Pro398 (σ), Met400, His401, Lys409, Glu410, Phe411, Leu430, Gly431, Glu432, Ser464 (σ)	V (Å^3) = 96.768<br>S (Å^2) = 256.461<br>LS (Å^2) = 136.613<br>D (Å) = 9.46573<br>Ac = 5<br>Do = 6<br>Ar = 6	H = 0.702128<br>AR = 2<br>BR = 3<br>PR = 3<br>APR = 5<br>TR = 13
**6**	Chain A = Thr360 (Δ), Lys361, Val362, Ala363, Cys375, Gly442, Met445, Lys446<br><br>Chain B = Asn456, Thr457, Ile458 (λ)	V (Å^3) = 75.776<br>S (Å^2) = 219.906<br>LS (Å^2) = 133.735<br>D (Å) = 6.88186<br>Ac = 7<br>Do = 5<br>Ar = 0	H = 0.690909<br>AR = 1<br>BR = 2<br>PR = 5<br>APR = 4<br>TR = 12

**7**	Chain A = Pro398 (σ), Met400, His401, Lys409, Glu410, Phe411, Gly431, Glu 432, Ser464 (σ)<br><br>Chain B = None	V (Å^3) = 64.00<br>S (Å^2) = 133.469<br>LS (Å^2) = 86.5001<br>D (Å) = 6.6453<br>Ac = 5<br>Do = 2<br>Ar = 6	H = 0.75<br>AR = 2<br>BR = 2<br>PR = 2<br>APR = 3<br>TR = 9
**8**	Chain A = None<br><br>Chain B = Met70, Arg85, Glu86, Ser87, Leu88, Cys89, Asn208, Lys211, Pro212, Arg213	V (Å^3) = 62.464<br>S (Å^2) =224.333<br>LS (Å^2) = 127.808<br>D (Å) = 5.1225<br>Ac = 5<br>Do = 3<br>Ar = 0	H = 0.655172<br>AR = 1<br>BR = 3<br>PR = 3<br>APR = 3<br>TR = 10
**9**	Chain A = Lys361, Gly376 (Δ), Leu377 (Δ), Tyr386, Glu423, Val424, Leu425, Met445<br><br>Chain B = None	V (Å^3) = 60.928<br>S (Å^2) = 246.745<br>LS (Å^2) = 118.111<br>D (Å) = 5.87878<br>Ac = 6<br>Do = 2<br>Ar = 0	H = 0.708333<br>AR = 1<br>BR = 1<br>PR = 2<br>APR = 4<br>TR = 8
**10**	Chain A = Phe396 (σ), Pro398 (σ), Leu399 (σ), Pro462 (σ), Thr463 (σ), Ser464 (σ)<br><br>Chain B = Lys61 (σ)	V (Å^3) = 56.832<br>S (Å^2) = 166.722<br>LS (Å^2) = 102.168<br>D (Å) = 5.1225<br>Ac = 2<br>Do = 3<br>Ar = 2	H = 0.761905<br>AR = 0<br>BR = 1<br>PR = 2<br>APR = 4<br>TR = 7
**11**	Chain A = None<br><br>Chain B = Asp71, Arg74, Gln241, Ala244, Asn245, Gly405, Ser406, Tyr408, Lys409	V (Å^3) = 49.152<br>S (Å^2) = 213.723<br>LS (Å^2) = 58.6415<br>D (Å) = 5.71314<br>Ac = 6<br>Do = 5<br>Ar = 4	H = 0.604651<br>AR = 1<br>BR = 2<br>PR = 5<br>APR = 1<br>TR = 9
**12**	Chain A = Gly13, Ser14, Leu17, Gly49, Cys52, Val53, Tyr110, Thr335, Ile339 (λ)<br><br>Chain B = None	V (Å^3) = 48.64<br>S (Å^2) = 131.502<br>LS (Å^2) = 98.3644<br>D (Å) = 4.93153<br>Ac = 5<br>Do = 3<br>Ar = 0	H = 0.714286<br>AR = 0<br>BR = 0<br>PR = 6<br>APR = 3<br>TR = 9
**13**	Chain A = Thr72, Glu75, Glu432<br><br>Chain B = Thr65, Gln68, Tyr69, Thr72	V (Å^3) = 37.888<br>S (Å^2) = 111.741<br>LS (Å^2) = 49.6961<br>D (Å) = 4.15692<br>Ac = 4<br>Do = 1<br>Ar = 2	H = 0.6<br>AR = 2<br>BR = 0<br>PR = 5<br>APR = 0<br>TR = 7

[1]Residues marked with yellow were not taken into account; [2]σ, λ, Δ = sigma, lambda and doorstop sites; [3]V = Volume; S = Surface; LS = LipoSurface; D = Depth; Ac = Number of solvent accessible hydrogen bond acceptors; Do = Number of solvent accessible hydrogen bond donors; Ar = Number of all aromatic atoms; H = Hydrophobicity of pocket; AR = Acidic residues; BR = Basic residues; PR = Polar residues; APR = Apolar residues; TR = Total residues.

Table S9. Composition of cavities found by Desmond in TRLMS.[1]

	Time (ns)		
**Cavity[2,3]**	**5**	**20**	**40**
**00**	Chain A = Ser14, Leu17, Glu18, Cys52, Val58, Lys61 (σ), Thr335, Pro336 (λ), Asn340 (λ), Gln439<br><br>Chain B = Phe396 (σ), Pro398 (σ), Leu399 (σ), Met400, His455, Asn456, Thr457, Ile458 (λ), Val460, His461 (λ), Pro462 (σ), Thr463 (σ), Ser464 (σ), Cys469, Arg472 (λ)<br><br>Area (Å^2) = 738.349<br>Sigma = 0.556	Chain A = Thr65, Gln68, Tyr69, Thr72, Glu75, Phe396 (σ), Pro398 (σ), Leu399 (σ), Met400, Asn402, Phe411, Glu432, Pro462 (σ), Thr463 (σ), Ser464 (σ)<br><br>Chain B = Lys61 (σ), Val64, Thr65, Gln68, Tyr69, Asp71, Thr72, Ile106, Phe367, Glu432<br><br>Area (Å^2) = 895.949<br>Sigma = 0.506	Chain A = Thr65, Gln68, Asp71, Thr72, Glu75, Asn245, Ile369, Phe396 (σ), Pro398 (σ), Leu399 (σ), Met400, His401, Asn402, Ser404, Lys407, Lys 409, Phe411, Glu432, Thr463 (σ), Ser464 (σ)<br><br>Chain B = Lys61 (σ), Val64, Thr65, Tyr69, Thr72, Ile106, Phe367, Glu432<br><br>Area (Å^2) = 1023.064<br>Sigma = 0.665
**01**	Chain A = Phe396 (σ), Pro398 (σ), Leu399 (σ), Thr457, Gly459 (λ), Val460, His461 (λ), Thr463 (σ), Ser464 (σ), Glu466 (λ), Cys469, Ser470 (λ), Arg472 (λ)<br><br>Chain B = Ser14, Leu17, Glu18, Trp21, Val53, Lys61 (σ), Ile106, Tyr110, Met113, Thr117, Pro336 (λ), Ile339 (λ), Asn340 (λ), Gln439<br><br>Area (Å^2) = 793.348<br>Sigma = 0.481	Chain A = His455, Ile458 (λ), Gly459 (λ), Val460, His461 (λ), Glu466 (λ), Cys469, Ser470 (λ), Arg472 (λ)<br><br>Chain B = Leu17, Glu18, Trp21, Val53, Pro336 (λ), Ile339 (λ), Asn340 (λ), Ala343 (λ), Gln439<br><br>Area (Å^2) = 475.859<br>Sigma = 0.479	Chain A = Thr457, Gly459 (λ), Val460, His461 (λ), Cys469, Arg472 (λ)<br><br>Chain B = Leu17, Val53, Pro336 (λ), Ile339 (λ), Asn340 (λ), Glu347, Arg355<br><br>Area (Å^2) = 396.261<br>Sigma = 0.480
**02**	Chain A = None<br><br>Chain B = Tyr198 (Δ), Gly229 (Δ), Phe230 (Δ), Val332 (Δ), Leu334 (Δ), Lys361, Val362, Cys364 (Δ), Thr374 (Δ), Leu377 (Δ), Leu425<br><br>Area (Å^2) = 415.285<br>Sigma = 0.366	Chain A = None<br><br>Chain B = Tyr198 (Δ), Phe230 (Δ), Val332 (Δ), Met333 (Δ), Leu334 (Δ), Val337, Lys361, Val362, Cys364 (Δ), Thr374 (Δ), Leu377 (Δ), Leu425<br><br>Area (Å^2) = 490.085<br>Sigma = 0.468	Chain A = Ser14, Cys52, Lys61 (σ), Thr65, Thr335, Pro336 (λ), Ile339 (λ), Asn340 (λ)<br><br>Chain B = Phe396 (σ), Pro398 (σ), Leu399 (σ), Met400, Ile458 (λ), Gly459 (λ), Val460, His461 (λ), Pro462 (σ), Thr463 (σ), Ser464 (σ), Glu466 (λ), Cys469, Arg472 (λ)<br><br>Area (Å^2) = 572.346<br>Sigma = 0.445
**03**	Chain A = Thr65, Gln68, Thr72, Phe367, Glu432<br><br>Chain B = Thr65, Gln68, Tyr69, Asp71, Phe367, Glu432<br><br>Area (Å^2) = 487.182<br>Sigma = 0.331	Chain A = Ser14, Glu18, Cys52, Pro336 (λ), Ile339 (λ), Asn340 (λ)<br><br>Chain B = Gln439, Asn456, Thr457, Ile458 (λ), Gly459 (λ), Val460, His461 (λ), Glu466 (λ)<br><br>Area (Å^2) = 482.693<br>Sigma = 0.411	Chain A = None<br><br>Chain B = Tyr198 (Δ), Phe230 (Δ), Met333 (Δ), Leu334 (Δ), Thr360 (Δ), Lys361, Val362, Cys364 (Δ), Thr374 (Δ), Leu377 (Δ), Leu425<br><br>Area (Å^2) = 424.792<br>Sigma = 0.508
**04**	Chain A = Tyr198 (Δ), Phe230 (Δ), Val332 (Δ), Cys364 (Δ), Thr374 (Δ), Gly376 (Δ), Leu377 (Δ), Leu425<br><br>Chain B = None<br><br>Area (Å^2) = 430.508<br>Sigma = 0.305	Chain A = Lys61 (σ), Thr65, Glu436<br><br>Chain B = Phe396 (σ), Pro398 (σ), Leu399 (σ), Met400, Glu432, His461 (λ), Pro462 (σ), Ser464 (σ), Glu466 (λ)<br><br>Area (Å^2) = 303.950<br>Sigma = 0.324	

[1]Residues marked with yellow were not taken into account; [2]The order of the cavities also indicates their relevance; [3]σ, λ, Δ = sigma, lambda and doorstop sites.

Table S10. Composition of cavities found by Desmond in TRLMS and 2W0H.[1]

	Time (ns)	
**Cavity[2,3]**	**20 (TRLMS)**	**20 (2W0H)**
**00**	Chain A = Lys61 (σ), Val64, Gln68, Tyr69, Asp71, Arg74, Gln241, Asn245, Phe367, Pro371, Phe396 (σ), Pro398 (σ), Met400, Lys409, Phe411, Glu432, Ser433, Glu436, Ser464 (σ), Glu467<br><br>Chain B = Lys61 (σ), Gln68, Thr72, Ile206, Pro398 (σ), Leu399 (σ), Met400, His401, Glu432, Ser433, Glu436, Pro462 (σ), Thr463 (σ), Ser464 (σ)<br><br>Area (Å^2) = 1068.707<br>Sigma = 0.841	Chain A = Lys61 (σ), Val64, Gln68, Leu72, Phe367, Ile369, Phe396 (σ), Leu399 (σ), Met400, Asp432, Ser433, His461 (λ), Pro462 (σ), Thr463 (σ), Ser464 (σ), Glu466 (λ), Glu467<br><br>Chain B = Ser14, Leu17, Glu18, Trp21, Cys52, Val58, Lys61 (σ), Thr65, Gln68, Leu72, Tyr110, Ile339 (λ), Phe367, Ser368, Asp432, Glu436<br><br>Area (Å^2) = 693.544<br>Sigma = 0.461
**01[4]**	Chain A = None<br><br>Chain B = Leu44, Ala46, Ala47, Gly50, Thr51, Asn54, Val55, Phe126, Glu141, Thr160, Gly161, Ser162, Trp163, Thr165, Arg166, Asp172, Cys175, Thr177, Ser178, Asn179, Ile199, Leu283, Ile285, Arg287, Arg290, Leu294, Val328<br><br>Area (Å^2) = 635.910<br>Sigma = 0.541	Chain A = Val36, Leu44, Ala46, Thr51, Val55, Phe126, Glu141, Thr160, Gly161, Ser162, Trp163, Arg287, Arg290, Gln292, Ala293, Leu294<br><br>Chain B = None<br><br>Area (Å^2) = 343.220<br>Sigma = 0.402
**02**	Chain A = Ile458 (λ), Val460, His461 (λ), Glu466 (λ)<br><br>Chain B = Ser14, Glu18, Trp21, Cys52, Val53, Cys57, Val58, Ile106, Tyr110, Met113, Pro336 (λ), Ile339 (λ), Asn340 (λ), Ala343 (λ)<br><br>Area (Å^2) = 531.845<br>Sigma = 0.407	Chain A = Pro336 (λ), Ile339 (λ), Asn340 (λ), Gln439<br><br>Chain B = Ile458 (λ), Gly459 (λ), Val460, His461 (λ), Cys469, Ser470 (λ)<br><br>Area (Å^2) = 283.445<br>Sigma = 0.390
**03**	Chain A = Arg228 (Δ), Gly229 (Δ), Phe230 (Δ), Met333 (Δ), Leu334 (Δ), Thr374 (Δ), Gly376 (Δ), Leu377 (Δ), Thr378 (Δ), His428<br><br>Chain B = None<br><br>Area (Å^2) = 315.183<br>Sigma = 0.315	Chain A = Ile458 (λ), Gly459 (λ), Val460, Glu466 (λ), Arg472 (λ)<br><br>Chain B = Pro336 (λ), Ile339 (λ), Asn340 (λ), Ala343 (λ), Gln439<br><br>Area (Å^2) = 244.780<br>Sigma = 0.280
**04[5]**	Chain A = None<br><br>Chain B = Tyr198 (Δ), Gly229 (Δ), Phe230 (Δ), Arg331 (Δ), Met333 (Δ), Val337, Lys361, Val362, Cys364 (Δ), Thr374 (Δ)<br><br>Area (Å^2) = 335.359<br>Sigma = 0.271	Chain A = Met70, Ile73, Arg74, Lys89, Lys211<br><br>Chain B = None<br><br>Area (Å^2) = 144.201<br>Sigma = 0.310
**05**	Chain A = None<br><br>Chain B = Leu167, Val194, Gly195, Cys220, Tyr221, Arg222, Ser254, Ile285<br><br>Area (Å^2) = 116.607<br>Sigma = 0.426	Chain A = None<br><br>Chain B = Tyr198 (Δ), Gly229 (Δ), Phe230 (Δ), Val332 (Δ), Leu334 (Δ), Lys361, Val362, Cys364 (Δ), Thr374 (Δ), Cys375, Gly376 (Δ), Leu425<br><br>Area (Å^2) = 283.586<br>Sigma = 0.255

06		Chain A = Lys101, Val102<br><br>Chain B = Ser464 (σ), Phe396 (σ), Thr397, Pro398 (σ), Leu399 (σ), Asn402<br><br>Area (Å^2) = 224.091<br>Sigma = 0.260

[1]Residues marked with yellow were not taken into account; [2]The order of the cavities also indicates their relevance; [3]σ, λ, Δ = sigma, lambda and doorstop sites; [4]The cavity is not located in a relevant area of 2W0H and TRLMS; [5]The 2W0H cavity is not located in a relevant area of the target.

Table S11. Comparison of key residues in the TryR of various parasites.

	**PDB (residues)**			
**Zone/ Pocket[1,2]**	**TRLMS (*L. mexicana*)**	**2JK6 (*L. infantum*)**	**1BZL (*T. cruzi*)**	**2WBA (*T. brucei*)**
C.C.	Val102	Val102	Ala103	Ala102
C.C.	Gly105	Ser105	Asn106	Asp105
C.C.	Lys112	Ser112	Glu113	Gly112
C.C.	Glu347	Glu347	Asp347	Asp347
C.C.	Ala356	Ala356	Lys356	Lys356
C.C.	Asn402	Asn402	Lys402	Asn402
C.C.	His455	His455	Tyr455	Tyr455
C.C.	Asn456	Ser456	Asn456	Asn456
C.C./λ-site	Pro336	Pro336	Pro336	Pro336
C.C./λ-site	Asn340	Asn340	Asn340	Asn340
C.C./λ-site	Ala343	Ala343	Ala343	Ala343
C.C./λ-site	Ile458	Ile458	Ile458	Ile458
C.C./λ-site	Gly459	Gly459	Gly459	Gly459
C.S.	Cys52	Cys52	Cys53	Cys52
C.S.	Cys57	Cys57	Cys58	Cys57
C.S./γ-g.s./λ-site	His461	His461	His461	His461
γ-g.s.	Glu467	Glu467	Glu467	Glu467
γ-g.s./λ-site	Glu466	Glu466	Glu466	Glu466
H.C.	Trp21	Trp21	Trp22	Trp21
H.C.	Tyr110	Tyr110	Tyr111	Tyr110
H.C.	Met113	Met113	Met114	Met113
H.C./λ-site	Ile339	Ile339	Ile339	Ile339
Z-site/σ-site	Phe396	Phe396	Phe396	Phe396
Z-site/σ-site	Pro398	Pro398	Pro398	Pro398

Z-site/σ-site	Leu399	Leu399	Leu399	Leu399
Y-site/λ-site	Ser470	Ser470	Ser470	Ser470
Y-site/λ-site	Arg472	Arg472	Arg472	Arg472
18-g.s.	Glu18	Glu18	Glu19	Glu18
σ-site	Pro462	Pro462	Pro462	Pro462
σ-site	Thr463	Thr463	Thr463	Thr463
σ-site	Ser464	Ser464	Ser464	Ser464
N.S.	Arg166	Arg166	His166	Met166
N.S.	Leu167	Leu167	Pro168	Pro167
N.S.	Val288	Val288	Ser289	Ile288
N.S.	Lys361	Lys361	Arg361	Arg361
N.S.	Glu423	Glu423	Thr423	Thr423
N.S./Δ	Tyr198	Tyr198	Phe199	Phe198
N.S./Δ	Arg228	Arg228	Arg229	Arg228
N.S./Δ	Gly229	Gly229	Gly230	Gly229
N.S./Δ	Phe230	Phe230	Phe231	Phe230
N.S./Δ	Arg331	Arg331	Arg331	Arg331
N.S./Δ	Val332	Val332	Val332	Leu332
N.S./Δ	Met333	Met333	Met333	Met333
N.S./Δ	Leu334	Leu334	Leu334	Leu334
N.S./Δ	Thr360	Thr360	Thr360	Thr360
N.S./Δ	Cys364	Cys364	Ser364	Ser364
N.S./Δ	Ala365	Ala365	Ala365	Ala365
N.S./Δ	Val366	Val366	Val366	Val366
N.S./Δ	Thr374	Thr374	Thr374	Thr374
N.S./Δ	Gly376	Gly376	Gly376	Gly376
N.S./Δ	Leu377	Met377	Leu377	Leu377
N.S./Δ	Thr378	Thr378	Ile378	Ile378
N.S./Δ	Glu381	Glu381	Val381	Val381
N.S./Δ	Ile385	Asn385	Arg385	Glu385

[1]C.C. = Catalytic Cavity, C.S. = Catalytic Site, γ-g.s. = γ-glutamic site, H.C. = Hydrophobic Cleft, 18-g.s. = 18-glutamic site, N.S. = NADPH Site; [2]Δ = doorstop pocket; [3]The residues marked yellow are those that differ between the TryR sequences of the parasites.

Table S12. Comparison of key residues between TryR and GR (glutathione reductase).

	PDB (residues)	
**Zone/ Pocket[1,2]**	**TRLMS (*L. mexicana*)**	**3DJJ (*H. sapiens*)**
C.C.	Asn22	Arg38
C.C.	Val102	Tyr106
C.C.	Gly105	Arg109
C.C.	Ile106	Leu110
C.C.	Ser109	Ile113
C.C.	Lys112	Asn116
C.C.	Ala344	Lys348
C.C.	Val346	Ala350
C.C.	Glu347	His351
C.C.	Arg355	Asp359
C.C.	Ala356	Ser360
C.C.	Thr357	Lys361
C.C.	His359	Tyr364
C.C.	Ser394	Thr401
C.C.	Asn402	Ala409
C.C.	Ser452	Ala458
C.C.	His455	Asp461
C.C.	Val460	Ile466
C.C./λ-site	Pro336	Pro340
C.C./λ-site	Asn340	Ala344
C.C./λ-site	Ala343	Arg347
C.C./λ-site	Ile458	Val464
C.C./λ-site	Gly459	Ala465
C.S.	Cys52	Cys58
C.S.	Cys57	Cys63
C.S./γ-g.s./λ-site	His461	His467
γ-g.s./ λ-site	Glu466	Glu472
Y-site/ λ-site	Ser470	Thr476
Y-site/ λ-site	Arg472	n/a
γ-g.s.	Glu467	Glu473

H.C.	Trp21	Arg37
H.C.	Tyr110	Tyr114
H.C.	Met113	Asn117
H.C./λ-site	Ile339	Ile343
Z-site/σ-site	Phe396	Phe403
Z-site/σ-site	Pro398	Pro405
Z-site/σ-site	Leu399	Met406
18-g.s.	Glu18	Ala34
σ-site	Pro462	Pro468
σ-site	Thr463	Thr469
σ-site	Ser464	Ser470
N.S.	Thr165	His164
N.S.	Arg166	Glu165
N.S.	Leu167	Ser166
N.S.	Lys361	Asn366
N.S.	Ala363	Pro368
N.S.	Cys375	Val380
N.S.	Lys384	His389
N.S.	Gly422	Glu428
N.S.	Glu423	Lys429
N.S.	Val427	Ile433
N.S./Δ	Tyr198	Tyr197
N.S./Δ	Arg228	Arg224
N.S./Δ	Gly229	Ser225
N.S./Δ	Phe230	Phe226
N.S./Δ	Arg331	Lys335
N.S./Δ	Val332	Ala336
N.S./Δ	Met333	Leu337
N.S./Δ	Leu334	Leu338
N.S./Δ	Thr360	Asn365
N.S./Δ	Cys364	Thr369
N.S./Δ	Ala365	Val370
N.S./Δ	Val366	Val371

N.S./Δ	Thr374	Thr379
N.S./Δ	Gly376	Gly381
N.S./Δ	Leu377	Leu382
N.S./Δ	Thr378	Thr383
N.S./Δ	Glu381	Glu386
N.S./Δ	Ile385	Lys390

[1]C.C. = Catalytic Cavity, C.S. = Catalytic Site, γ-g.s. = γ-glutamic site, H.C. = Hydrophobic Cleft, 18-g.s. = 18-glutamic site, N.S. = NADPH Site; [2]Δ = doorstop pocket; [3]The residues marked yellow are those that differ between the TryR sequences of the parasites.

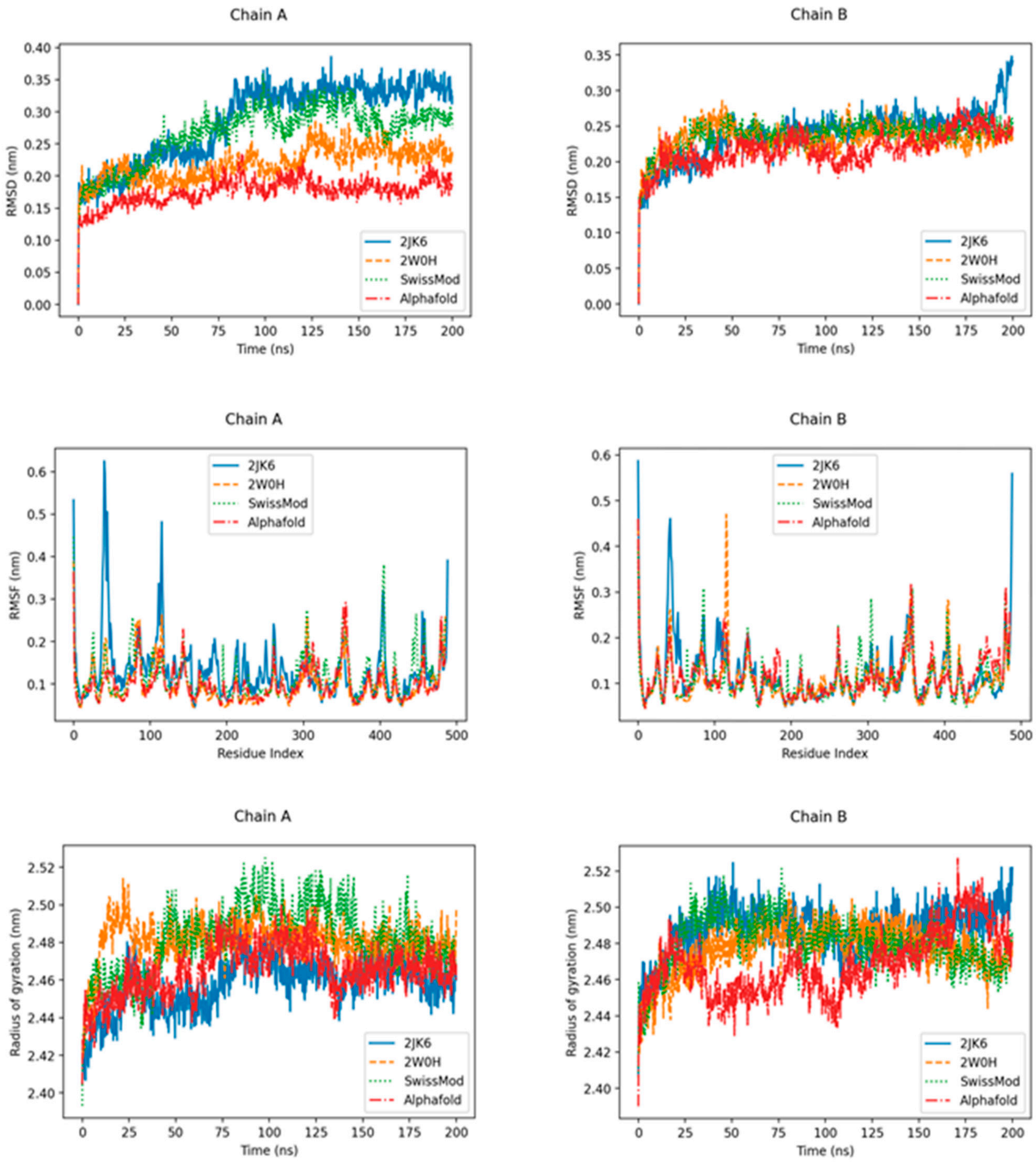

Figure S1. Comparative of Root-mean squared distance (RMSD; top), Root-mean square fluctuation (RMSF; middle) and Radius of gyration (bottom) of the two chains of TryR in simulated systems.

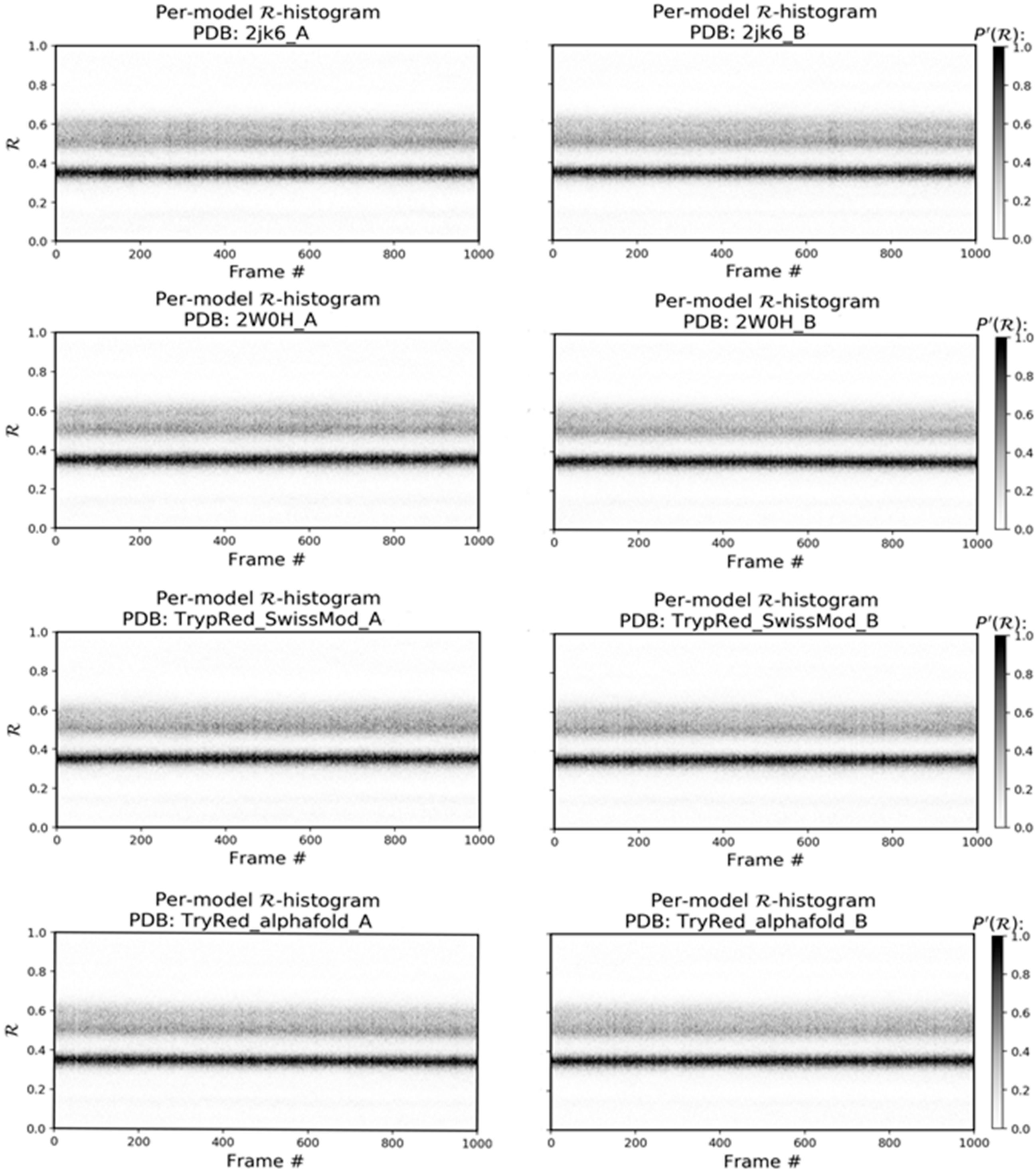

Figure S2. Ramachandran number histograms, as obtained with Backmap for the simulated systems.

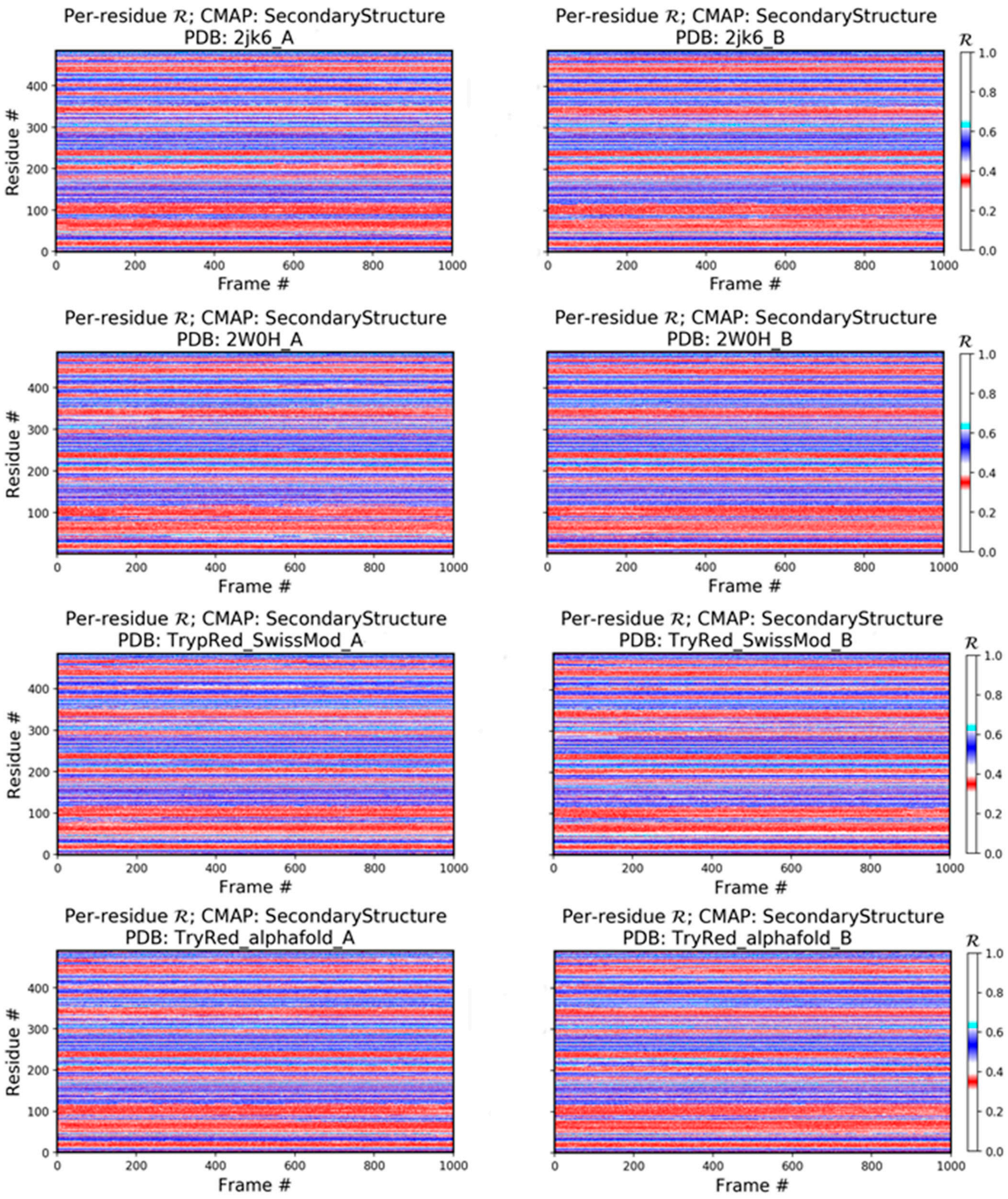

Figure S3. Secondary structure distribution, as obtained with Backmap for the simulated systems.

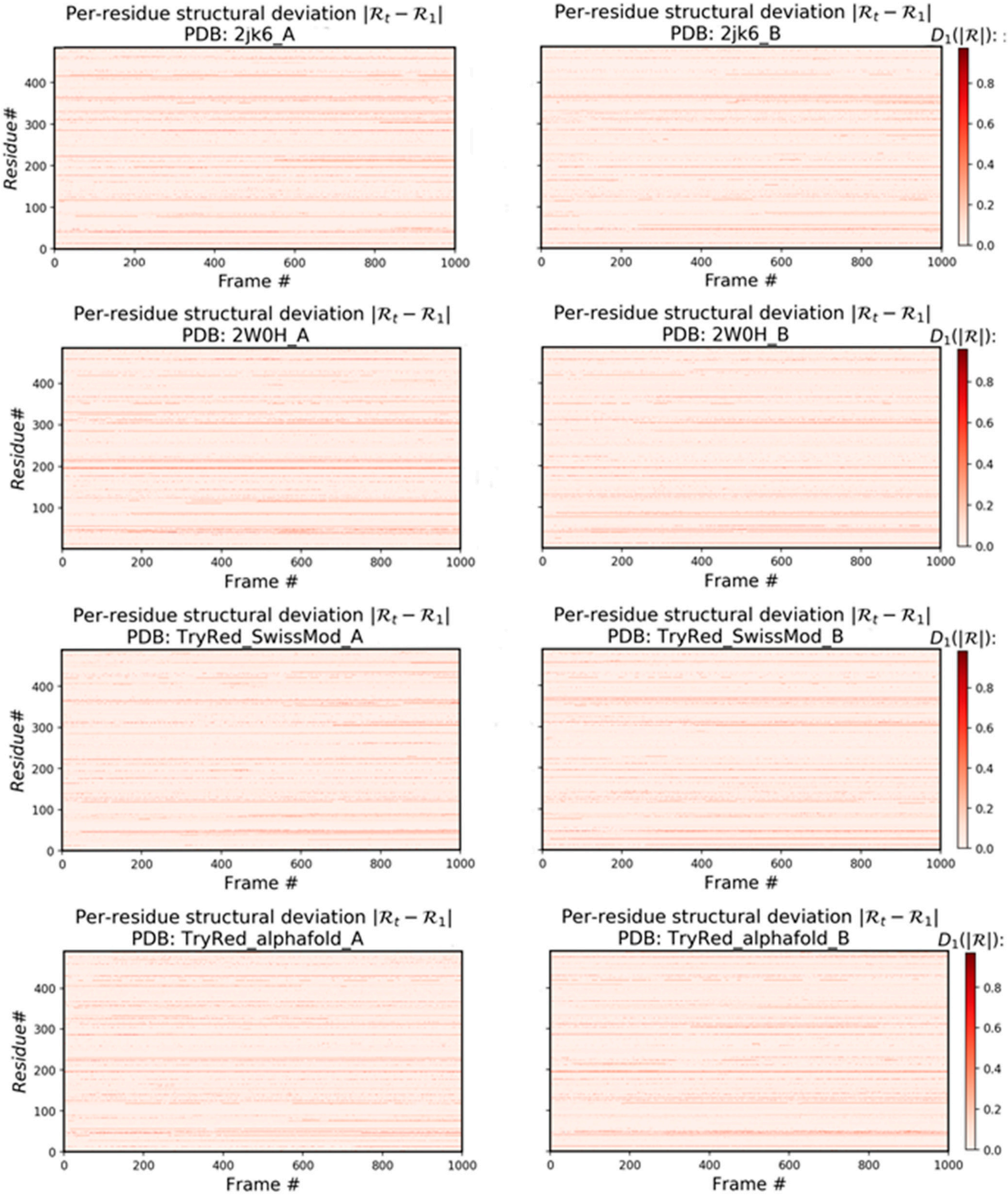

Figure S4. Secondary structure deviation, as obtained with Backmap for the simulated systems.

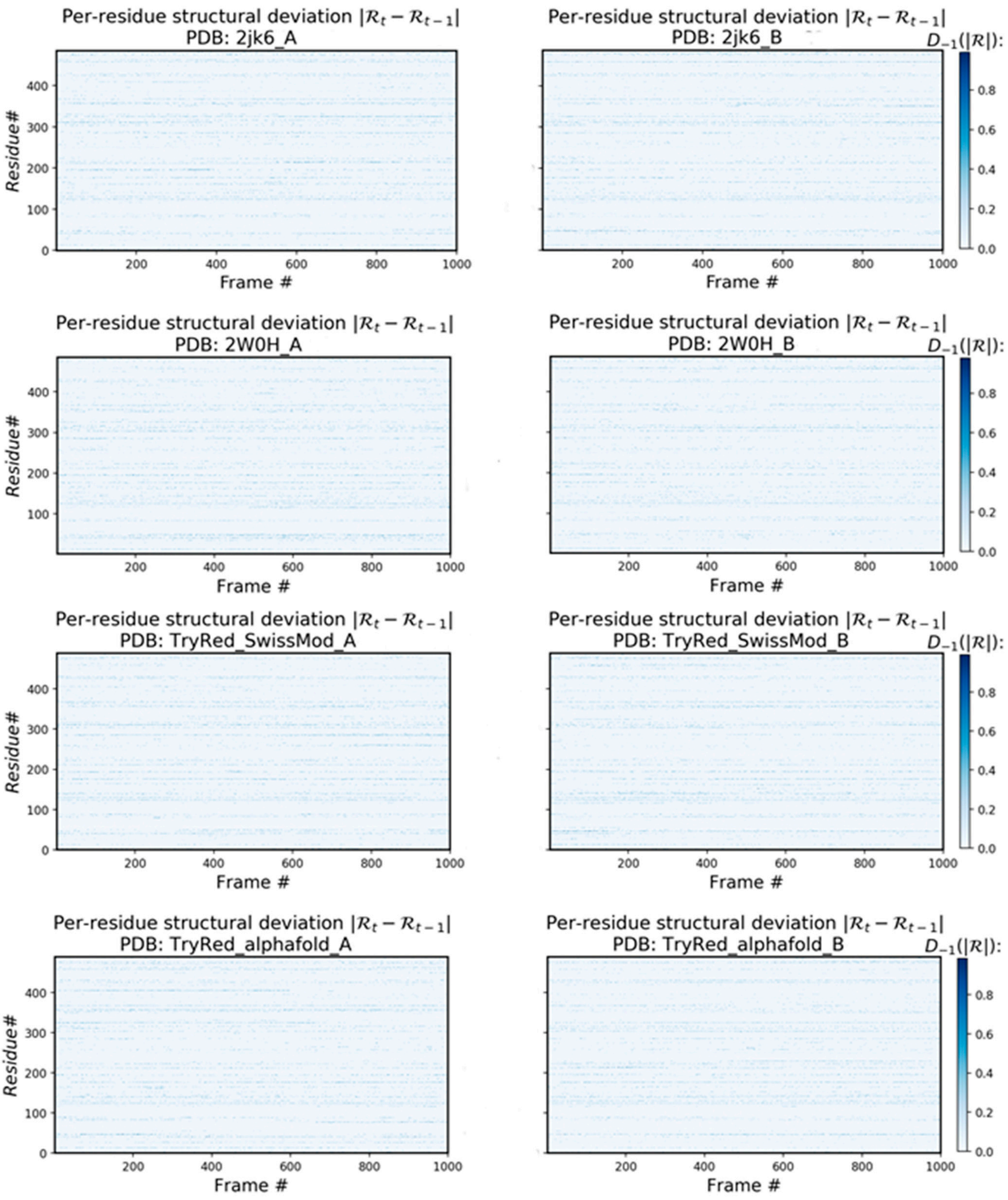

Figure S5. Secondary structure fluctuation, as obtained with Backmap for the simulated systems.

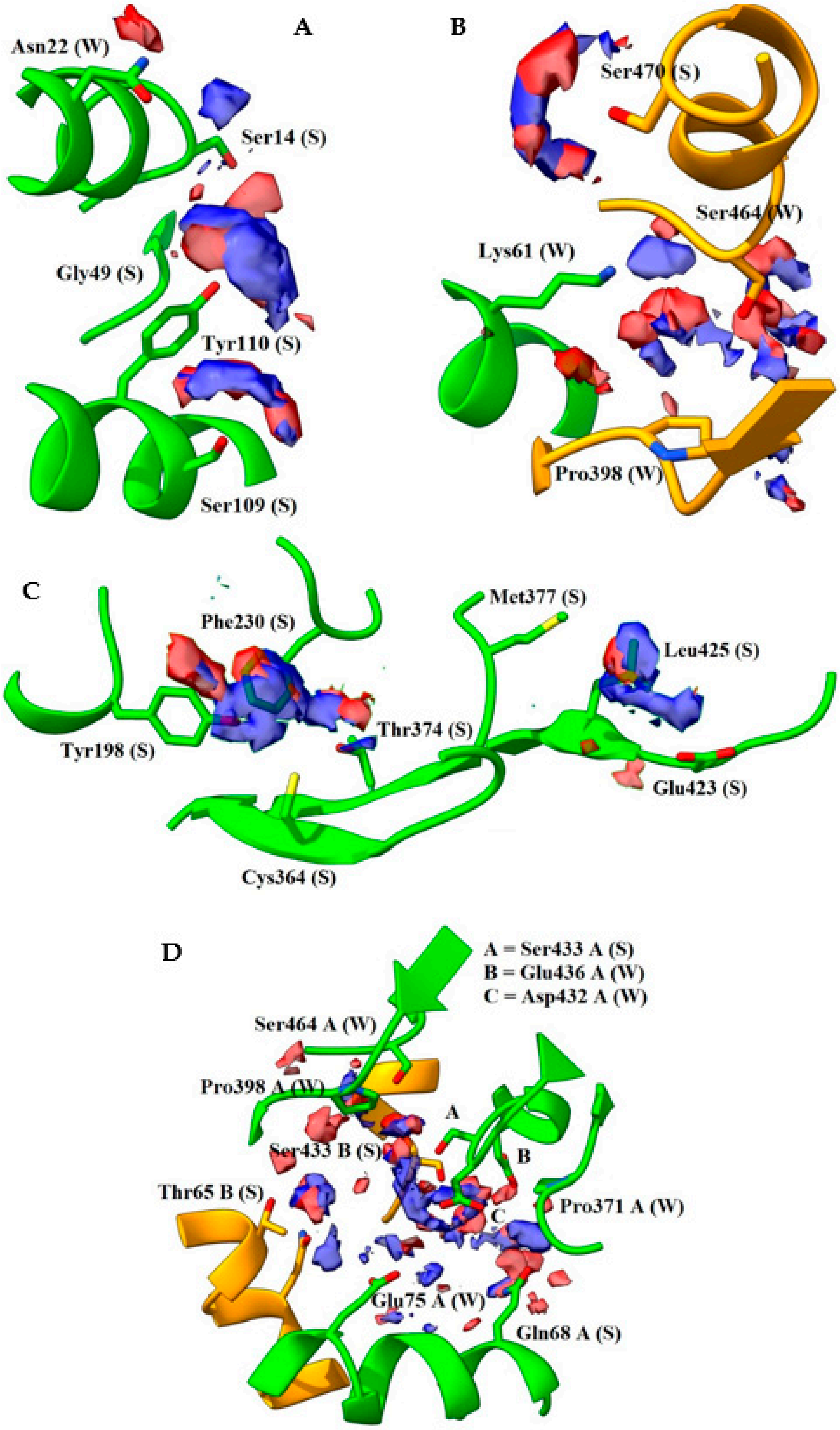

Figure S6. Residues marked as hot spots by FHM in 2W0H. The residues considered as strong hot spots are marked with (S) and the weak hot spots with (W). FHM shows the hotspots as surfaces of an electron donor (blue) or acceptor (red) character. (A) and (B) = catalytic cavity; (C) = NADPH site; (D) = interface.

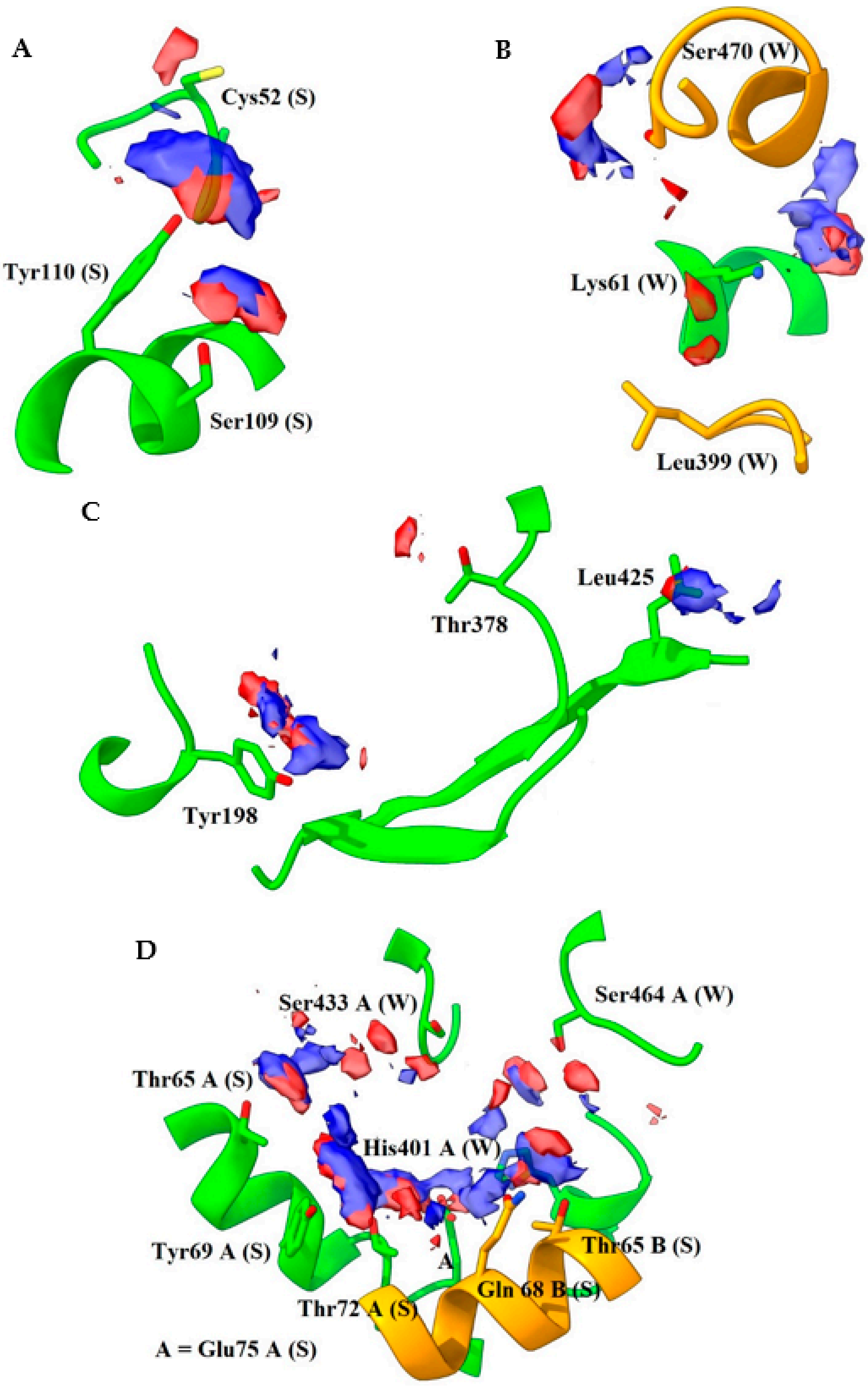

Figure S7. Residues marked as hot spots by FHM in TRLMS. The residues considered as strong hot spots are marked with (S) and the weak hot spots with (W). FHM shows the hotspots as surfaces of an electron donor (blue) or acceptor (red) character. (A) and (B) = catalytic cavity; (C) = NADPH site; (D) = interface.

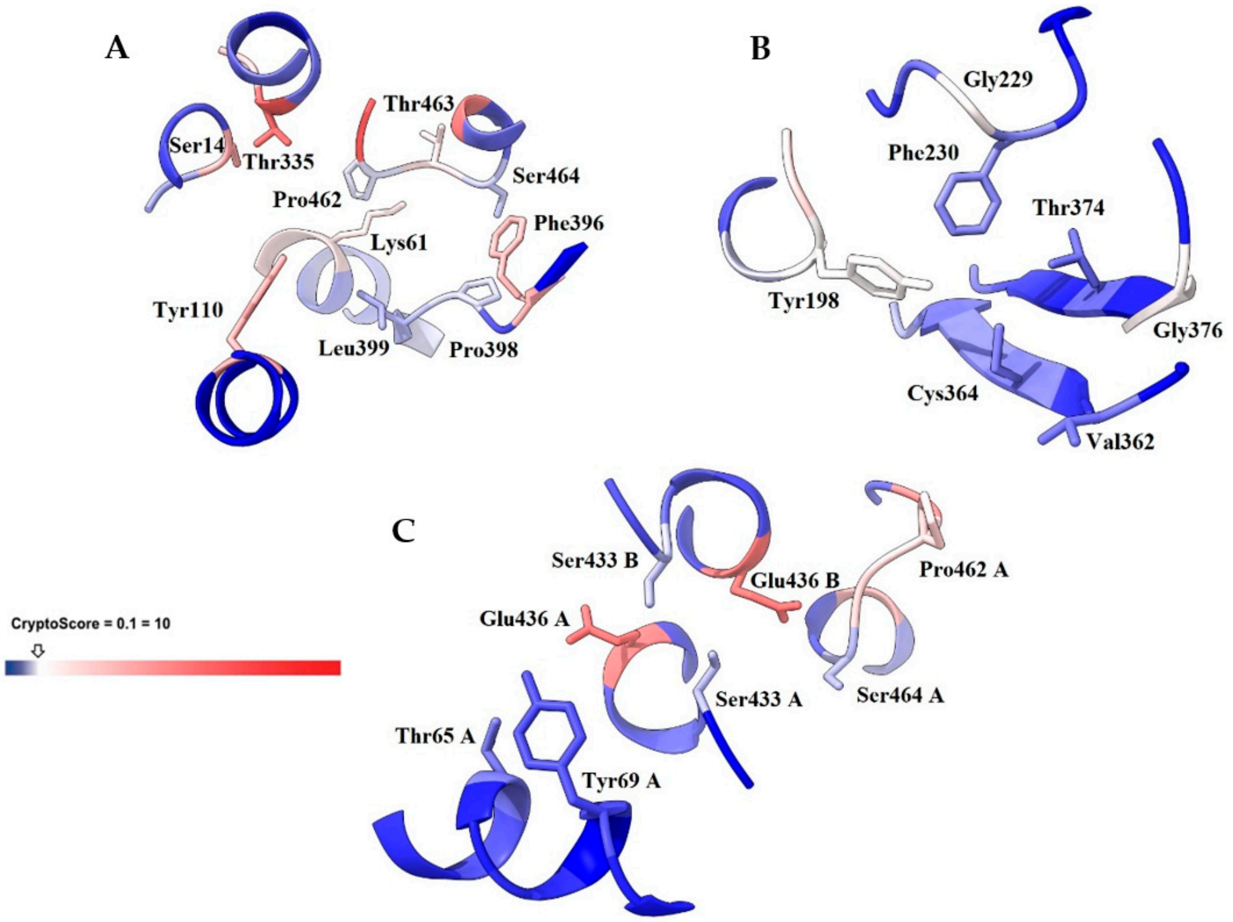

Figure S8. Residues marked as cryptic sites by CryptoSite in 2W0H. (A) = catalytic cavity; (B) = NADPH site; (C) = interface. Review Table S6.

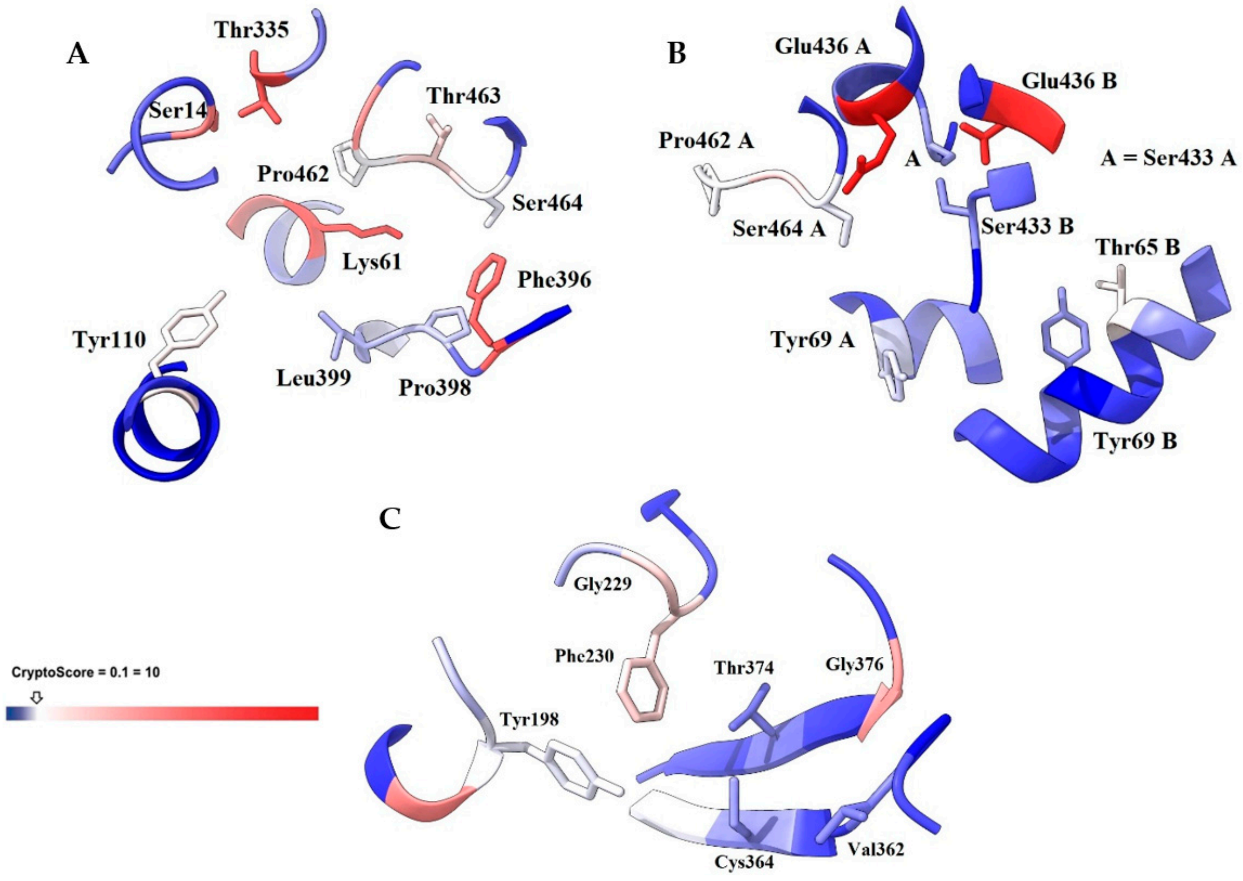

Figure S9. Residues marked as cryptic sites by CryptoSite in TRLMS. (A) = catalytic cavity; (B) = interface; (C) = NADPH site. Review Table S6.

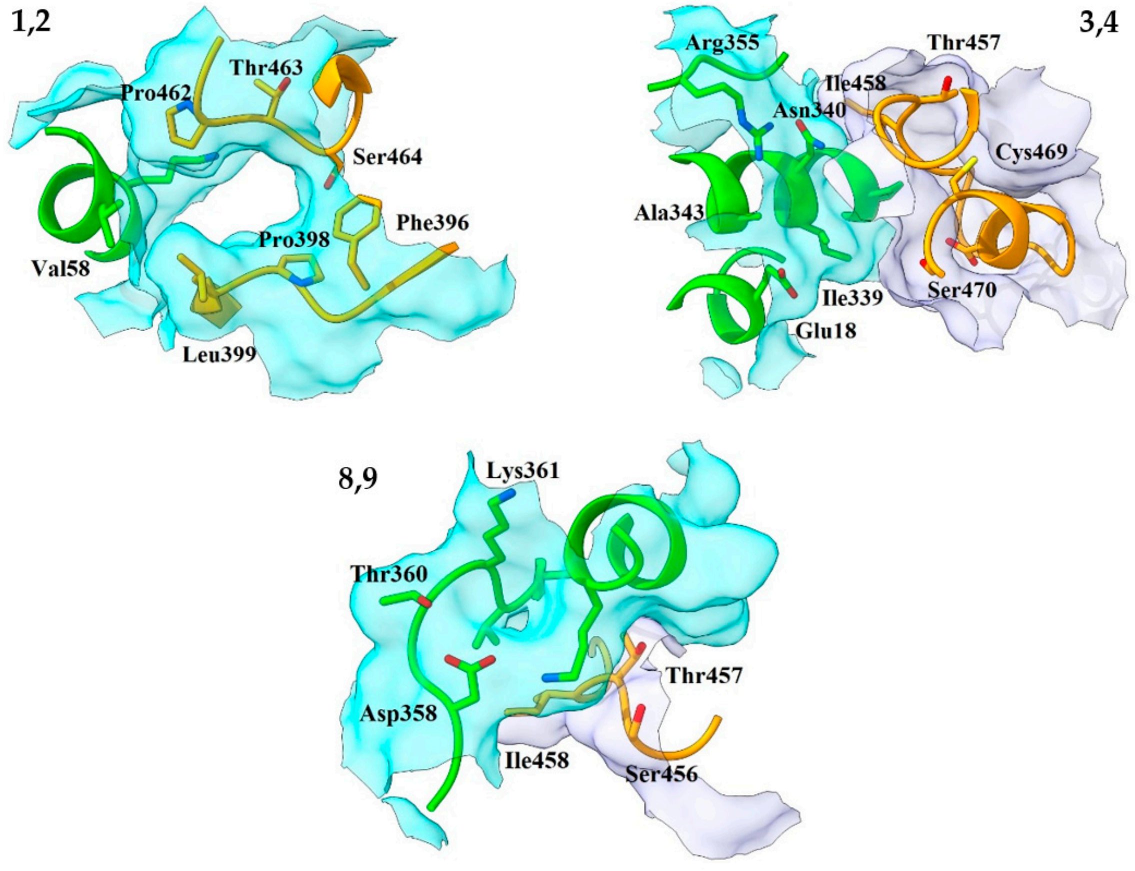

Figure S10. Most representative cavities found by DogSite3 in 2W0H. The number marks the cavity number and its relevance (Table S7).

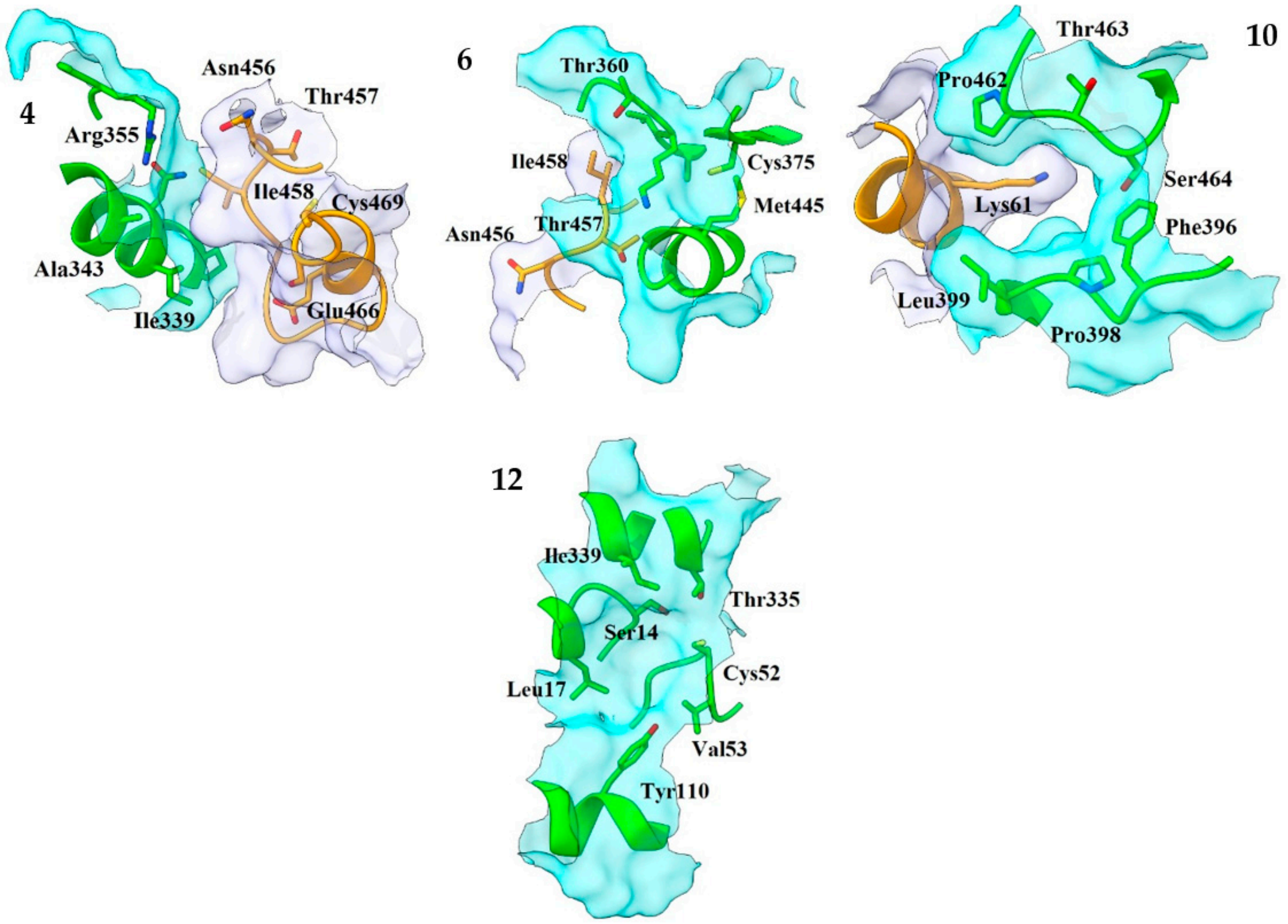

Figure S11. Most representative cavities found by DogSite3 in TRLMS. The number marks the cavity number and its relevance (Table S8).

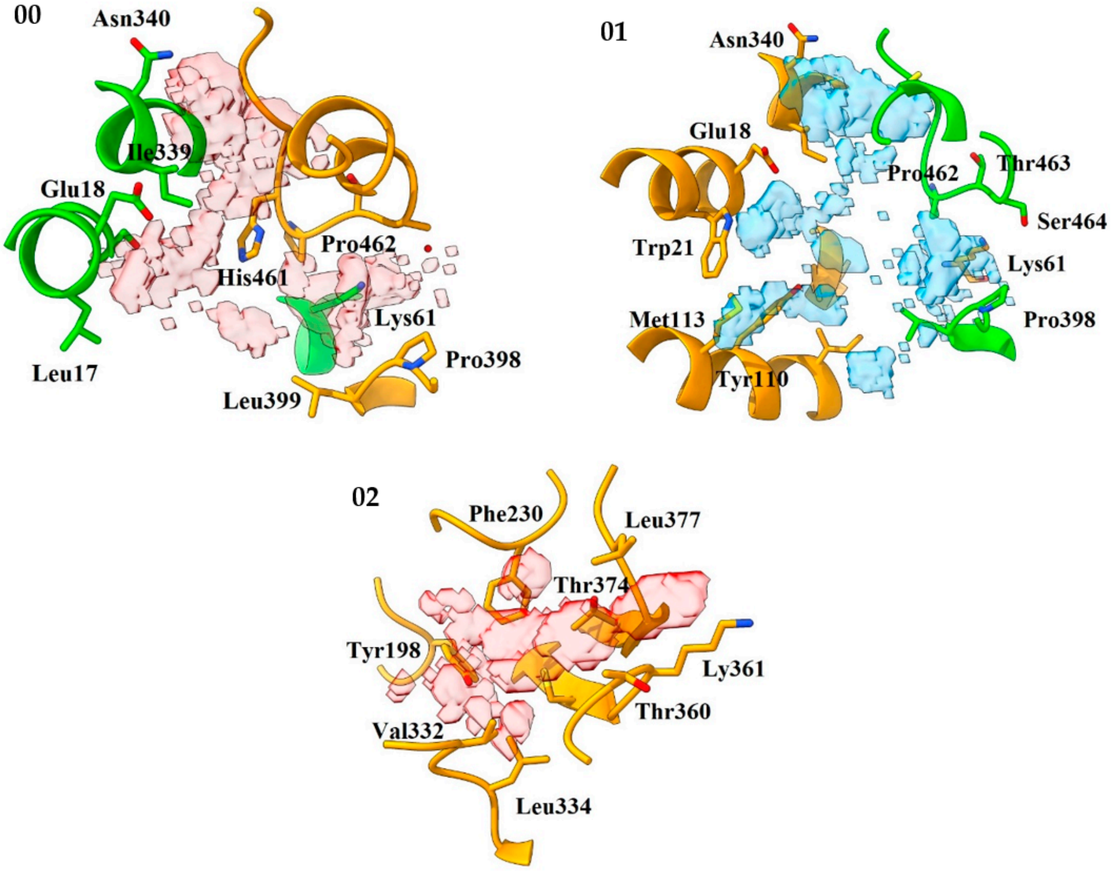

Figure S12. Most representative cavities found by Desmond in TRLMS (5 ns, Experiment A). The number indicates the cavity and its relevance (Table S9).

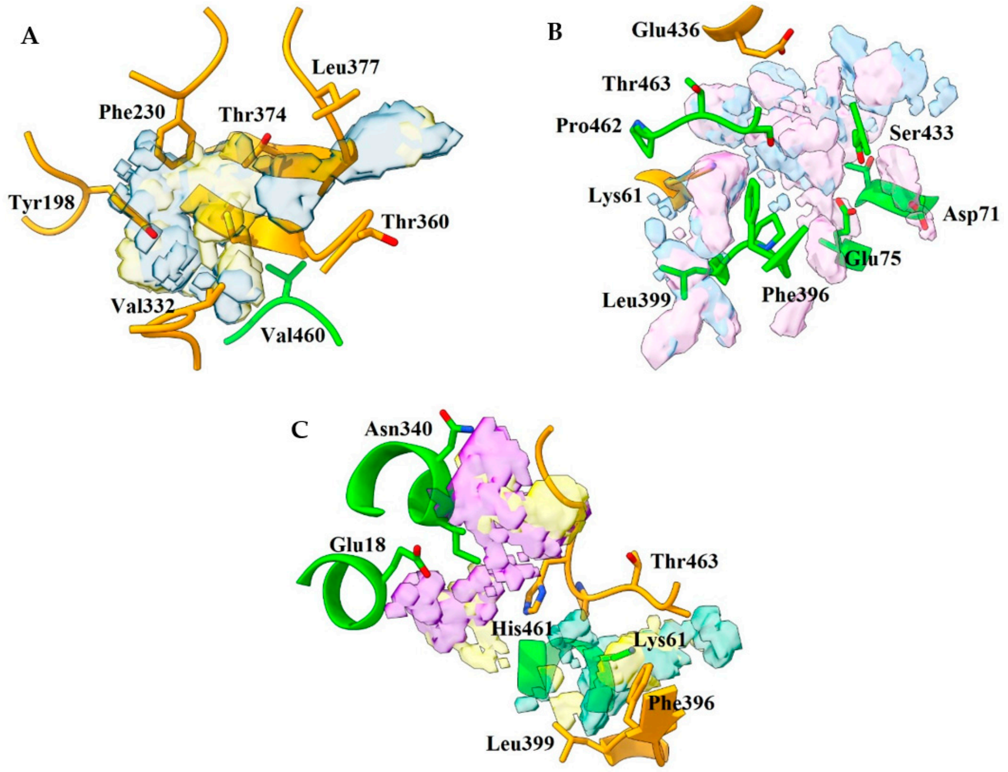

Figure S13. Most representative cavities found by Desmond in TRLMS (20 and 40 ns, Experiment A). The number indicates the cavity and its relevance (Table S9). (A) 20 ns, cavity 02 (dark green) and 40 ns, cavity 03 (yellow green); (B) 20 ns, cavity 00 (blue) and 40 ns, cavity 00 (purple); (C) 20 ns, cavity 03 (deep purple), 04 (green) and 40 ns, cavity 02 (yellow) (Table S9).

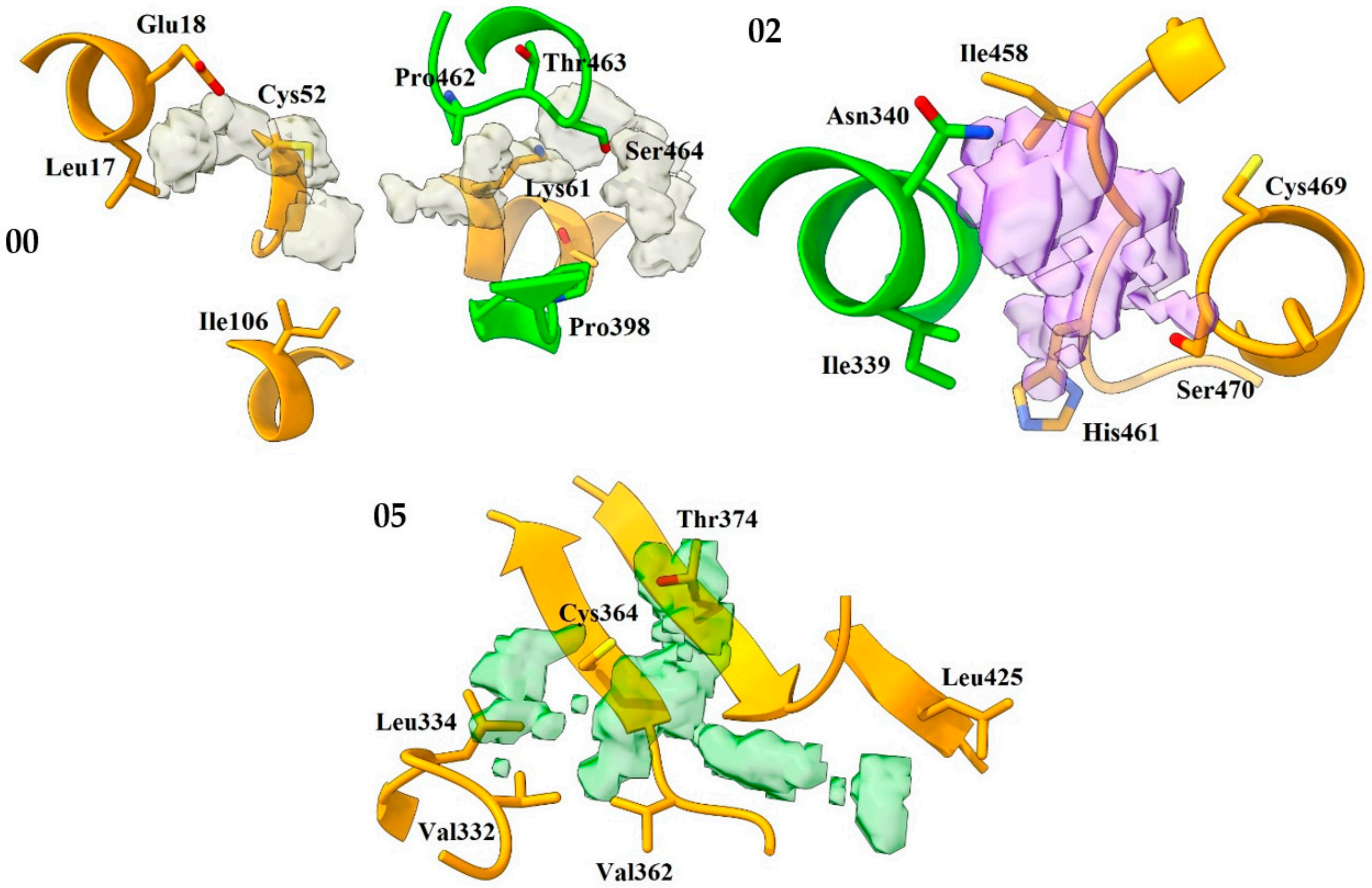

Figure S14. Most representative cavities found by Desmond in 2W0H (20 ns, Experiment B). The number indicates the cavity and its relevance (Table S10).

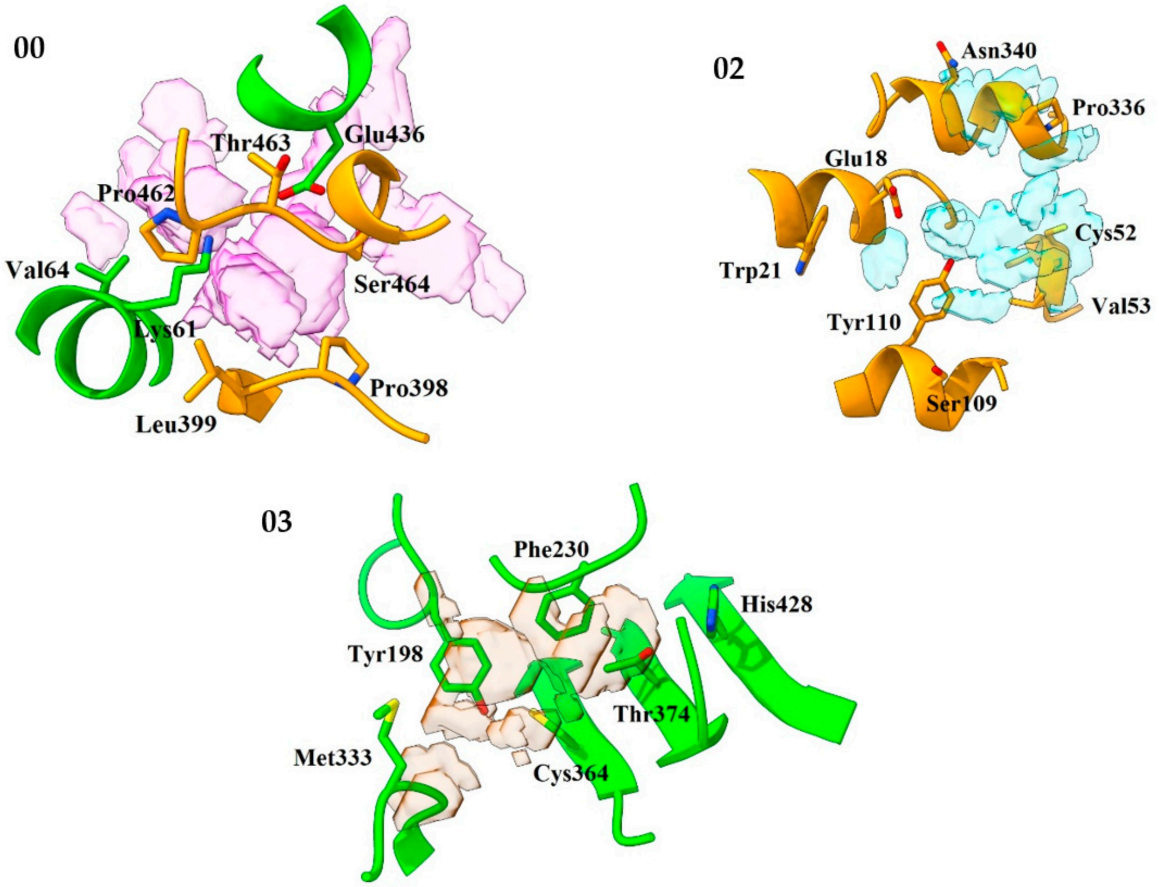

Figure S15. Most representative cavities found by Desmond in TRLMS (20 ns, Experiment C). The number indicates the cavity and its relevance (Table S10).

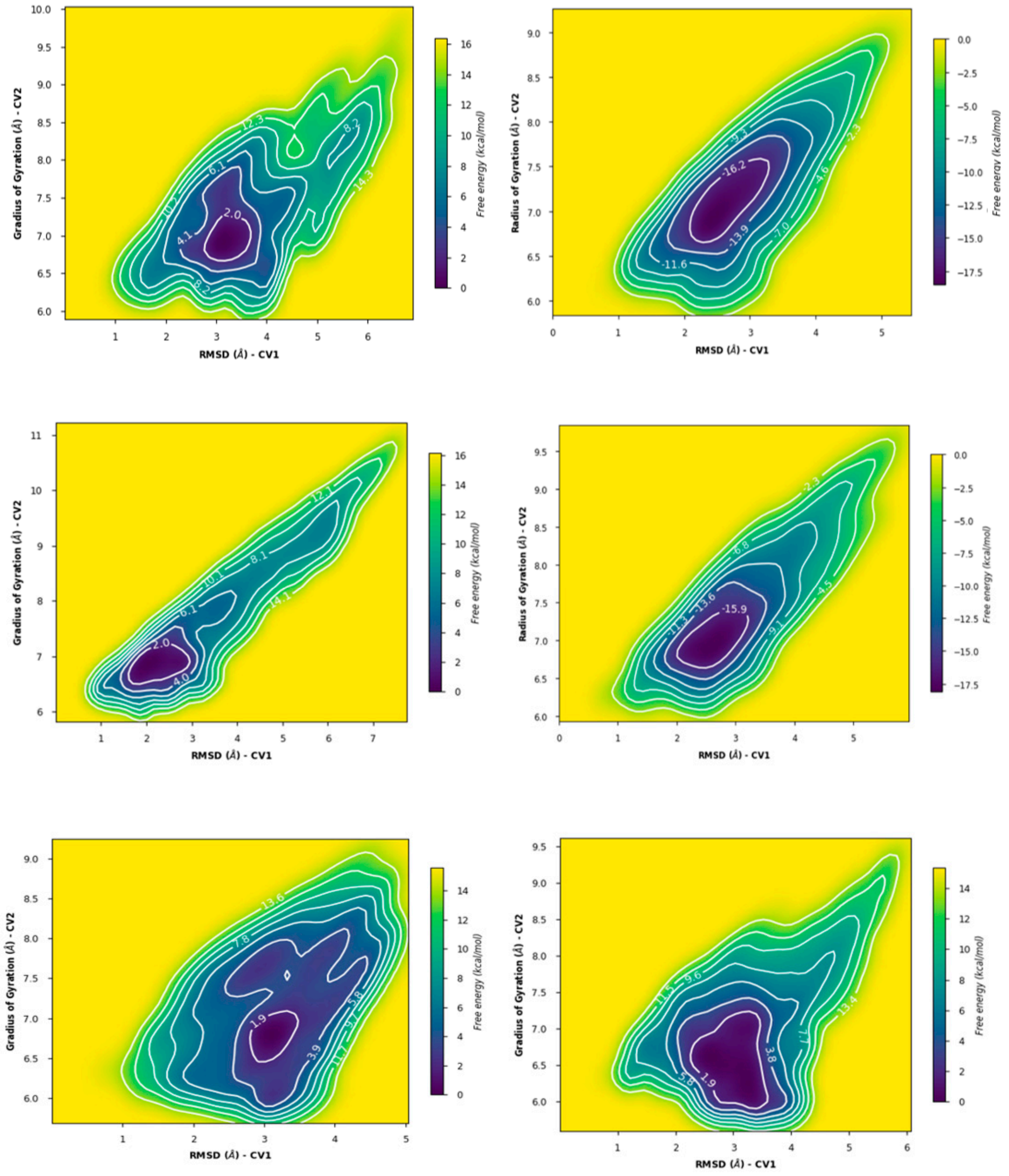

Figure S16. Free energy surfaces obtained from six independent runs of metadynamics simulations of 2W0H. Collective variables are RMSD and Radius of gyration, respectively.

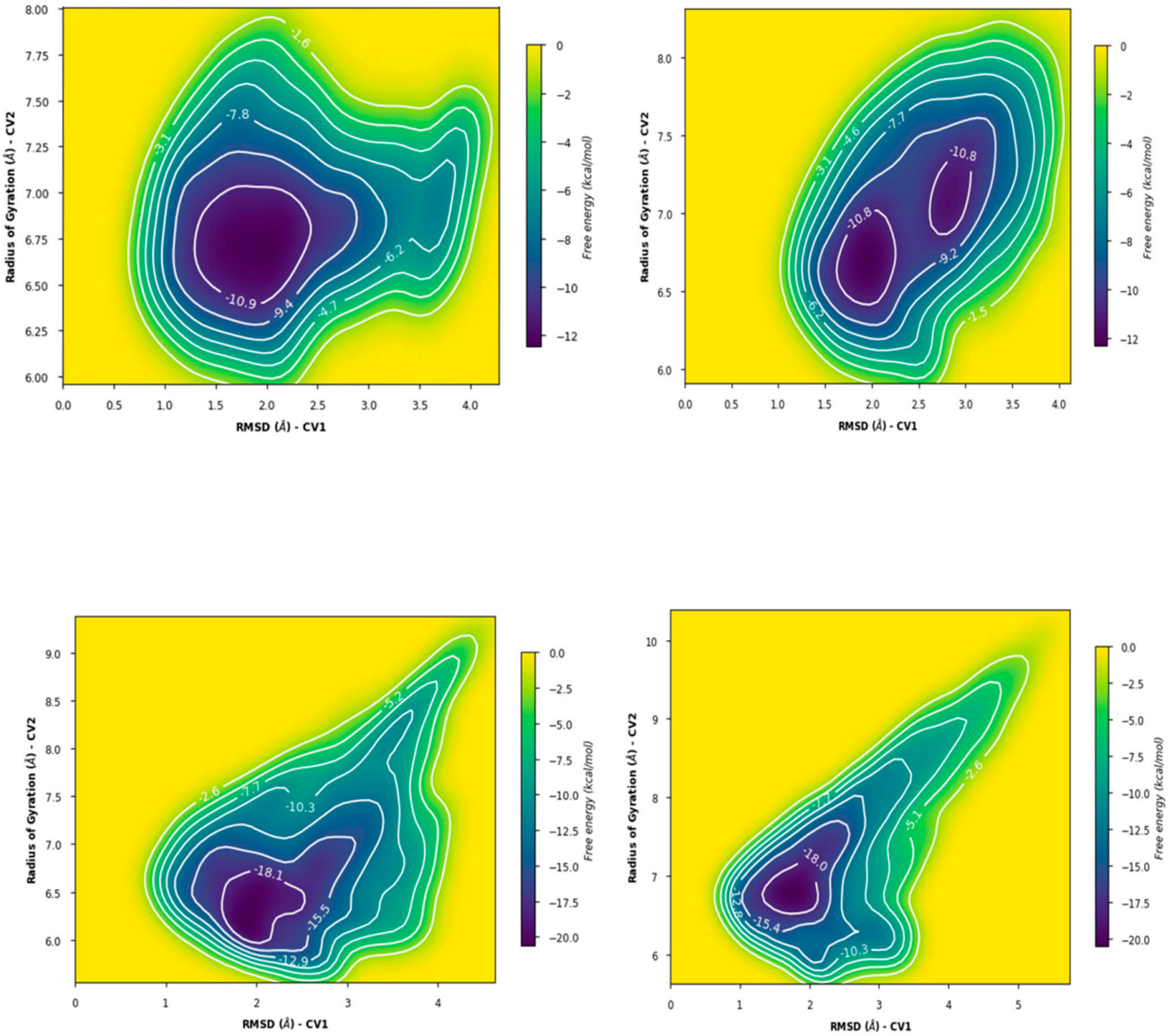

Figure S17. Free energy surfaces obtained from duplicate runs of metadynamics simulations of TRLMS. Collective variables are RMSD and Radius of gyration, respectively. Bias factor was of 3.0 (top) and 8.0 (bottom).

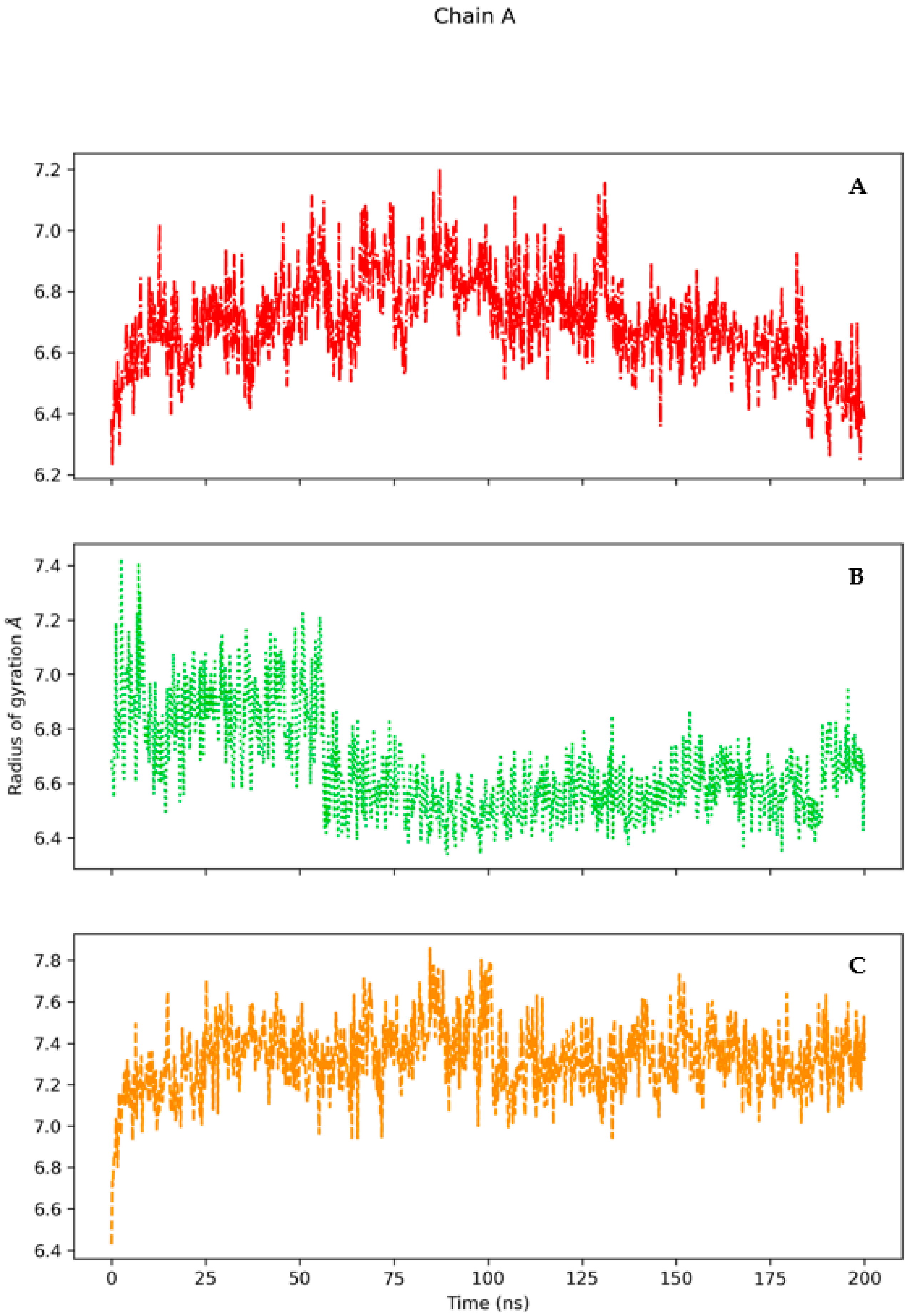

Figure S18. Radius of gyration plots as recovered from the initial simulations of homology models of TRLMA, TRLMS and 2W0H. Color scheme and line style are preserved from **Figure S1**. TRLMA = A; TRLMS = B; 2W0H = C.

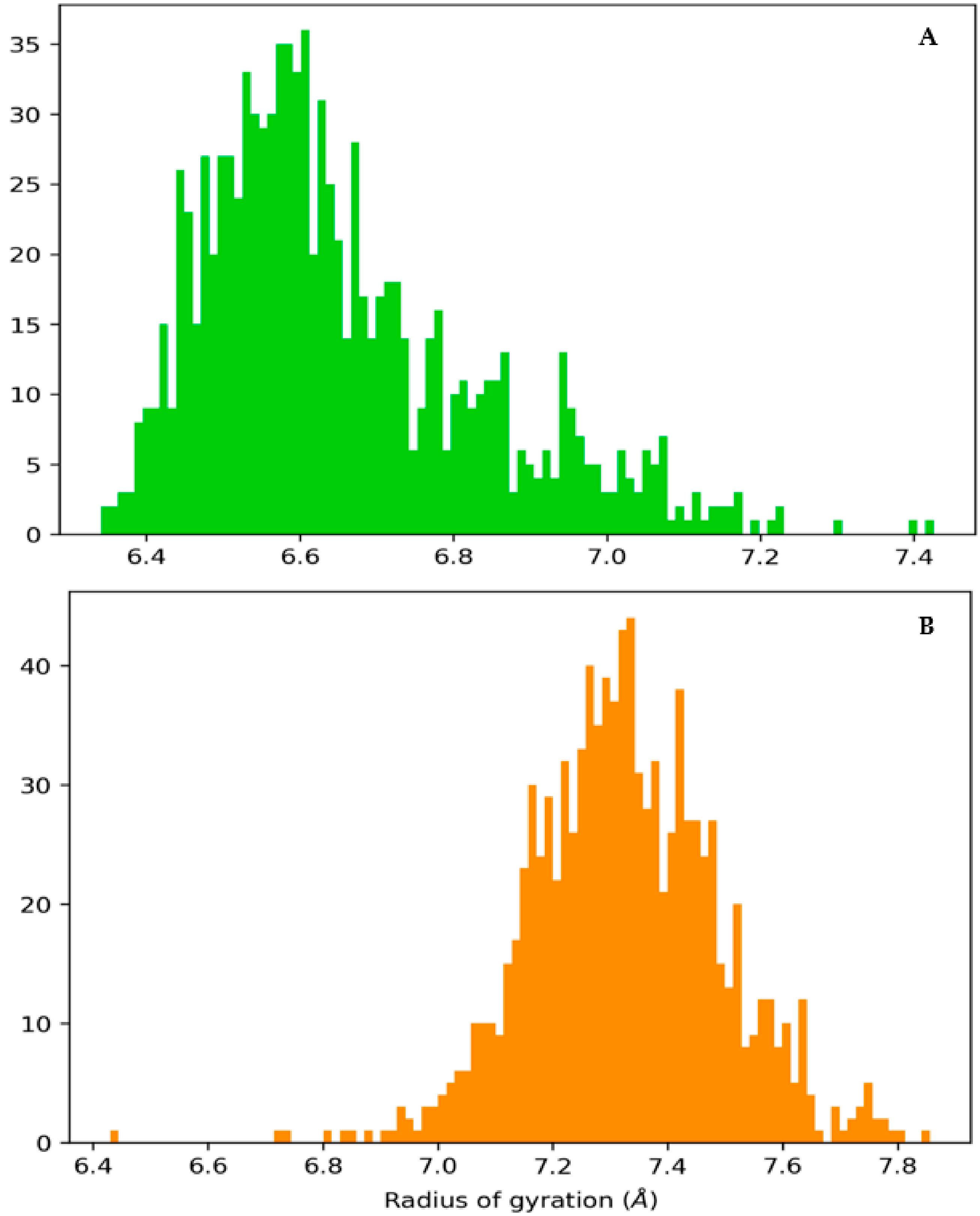

Figure S19. Histograms of radius of gyration values obtained from classical molecular dynamics simulations of TRLMS and 2W0H. Color scheme is preserved from **Figure S1**. TRLMS = A; 2W0H = B.

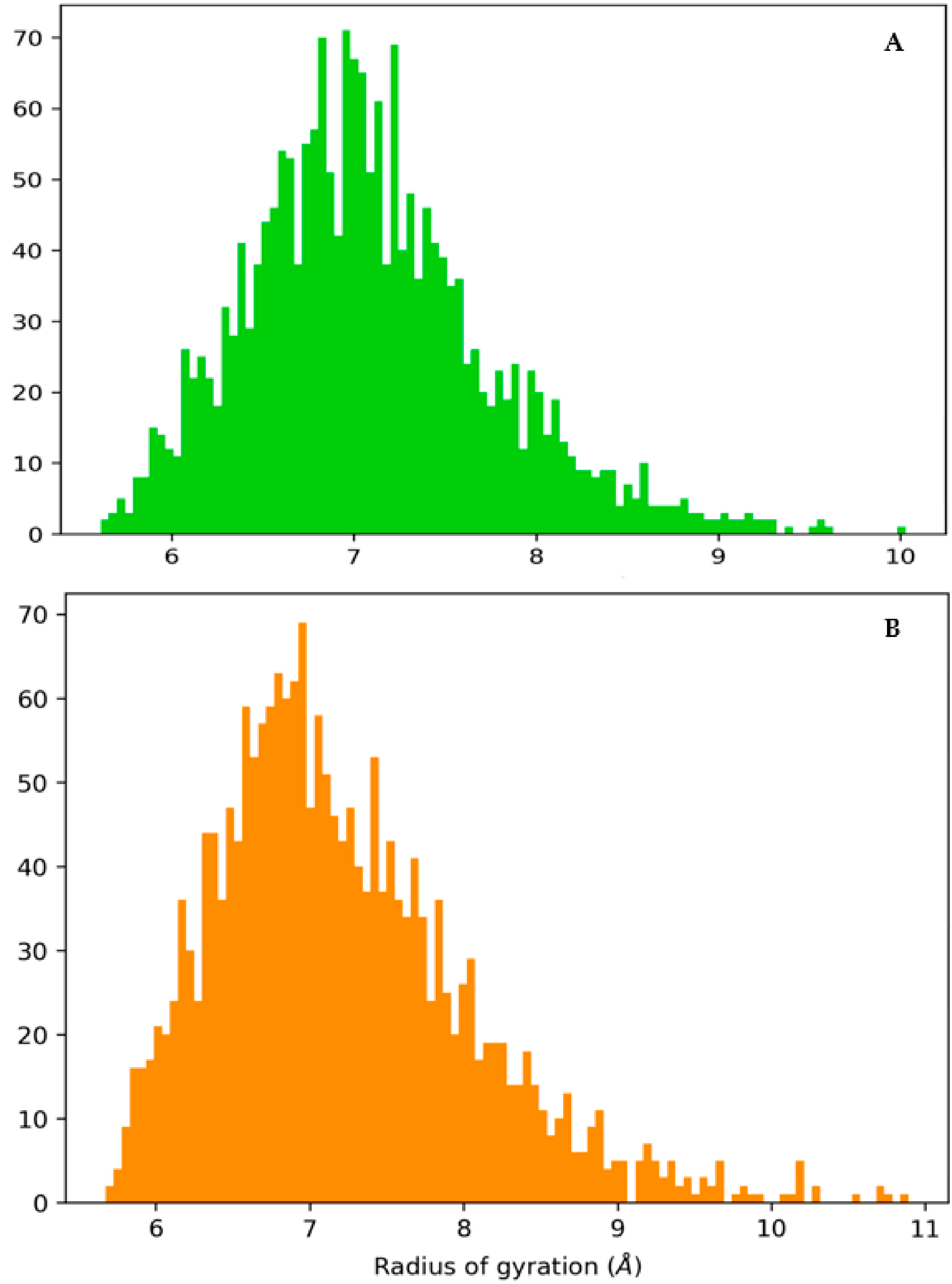

Figure S20. Histograms of radius of gyration values obtained from metadynamics simulations of TRLMS and 2W0H. Color scheme is preserved from **Figure S1**. TRLMS = A; 2W0H = B.

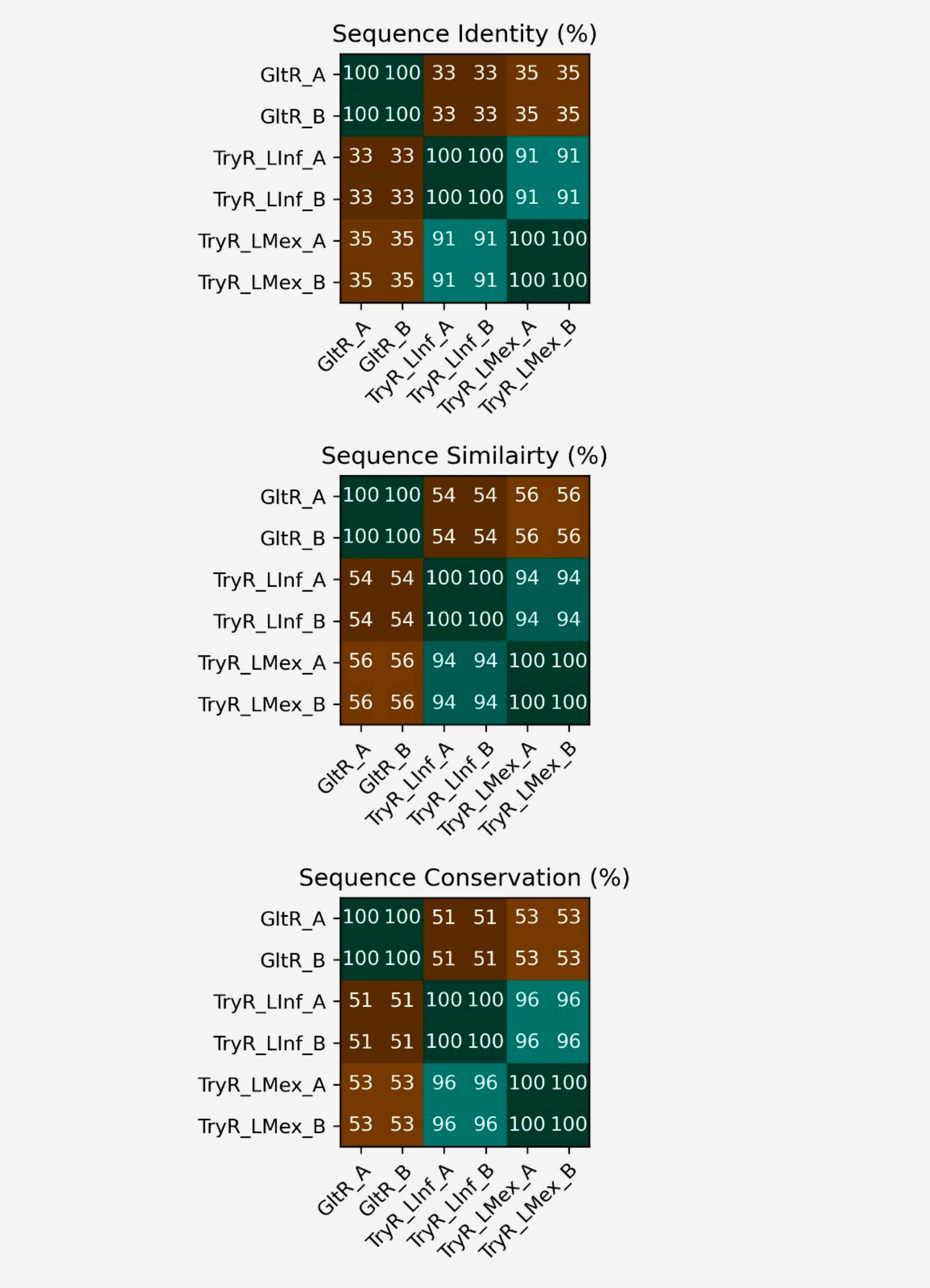

Figure S21. Sequence comparison between GR (3DJJ), TRLMS and 2JK6.

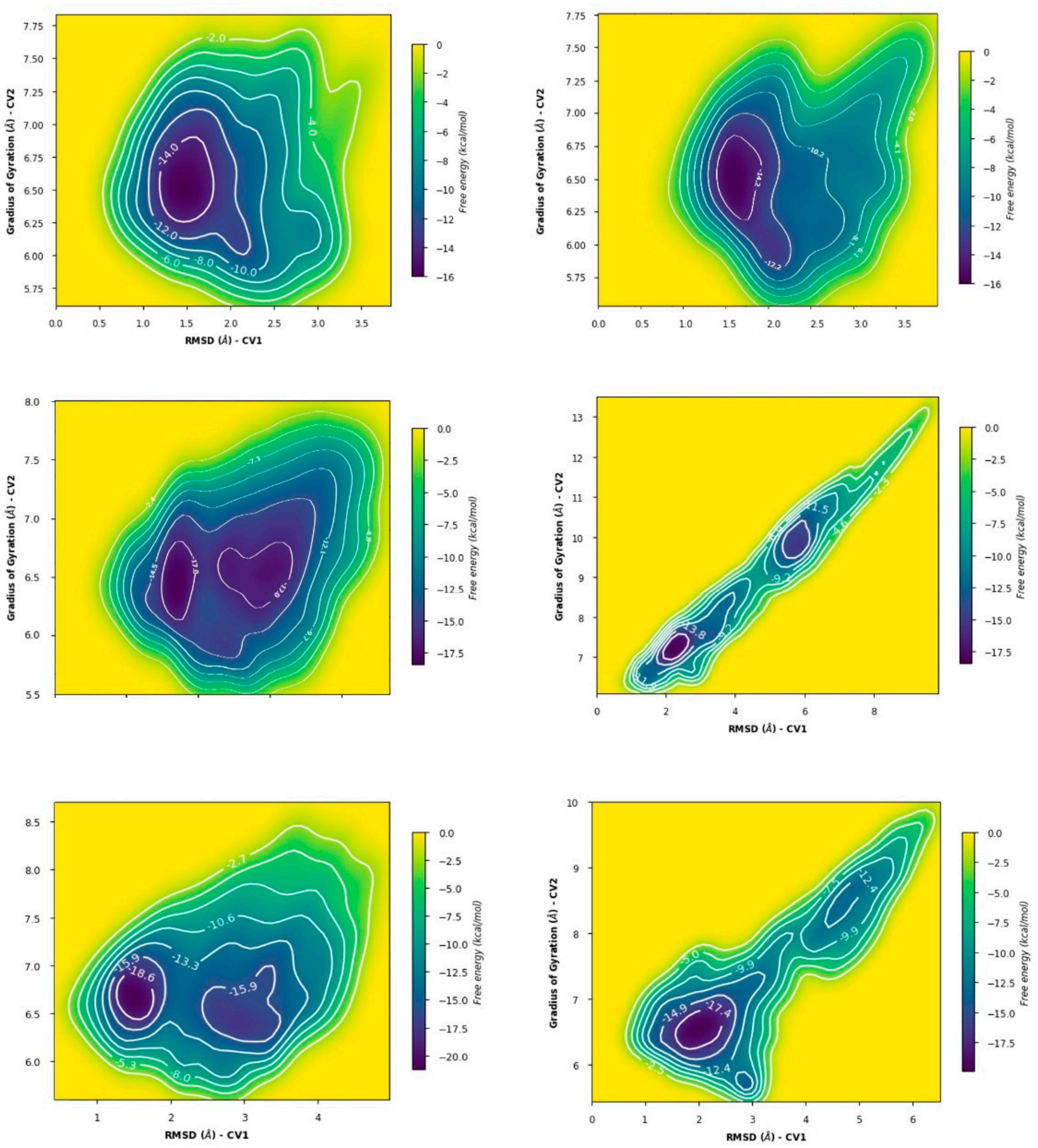

Figure S22. Free energy surfaces obtained from six independent runs of metadynamics simulations in GR for σ-site.

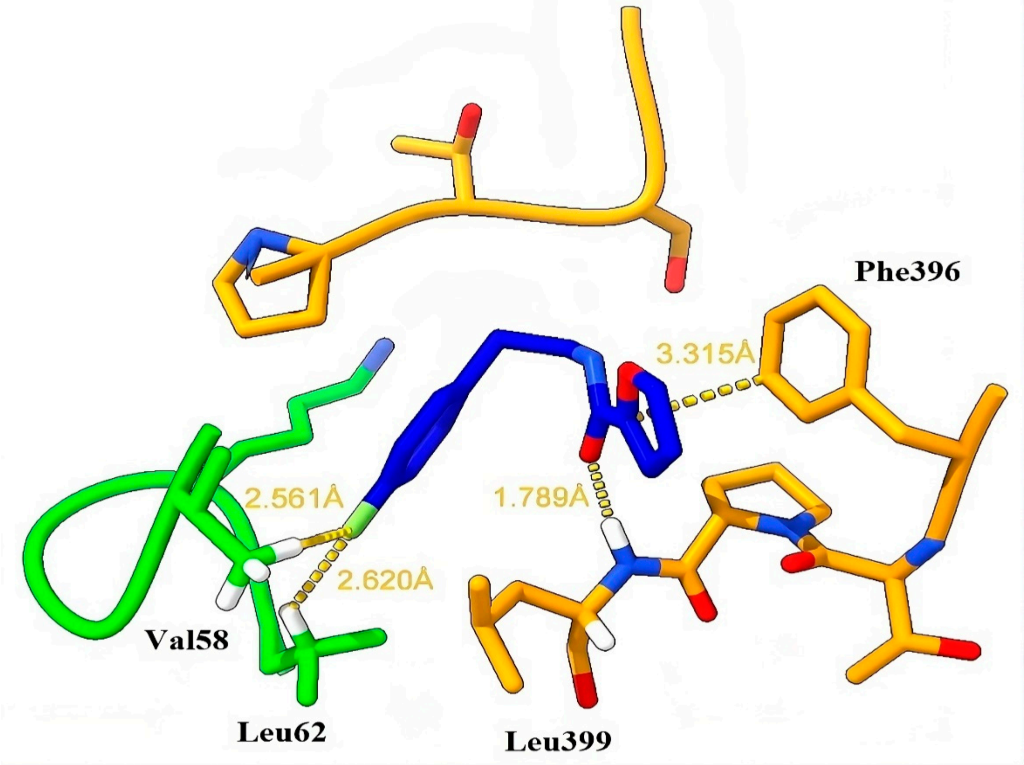

Figure S23. Native conformation of compound 71 in PDBID 5S9W. π-π interaction can be observed with 396 and a hydrogen bond with 399. Minor interactions are observed with residues 58 and 62. The ligand is represented with blue sticks.

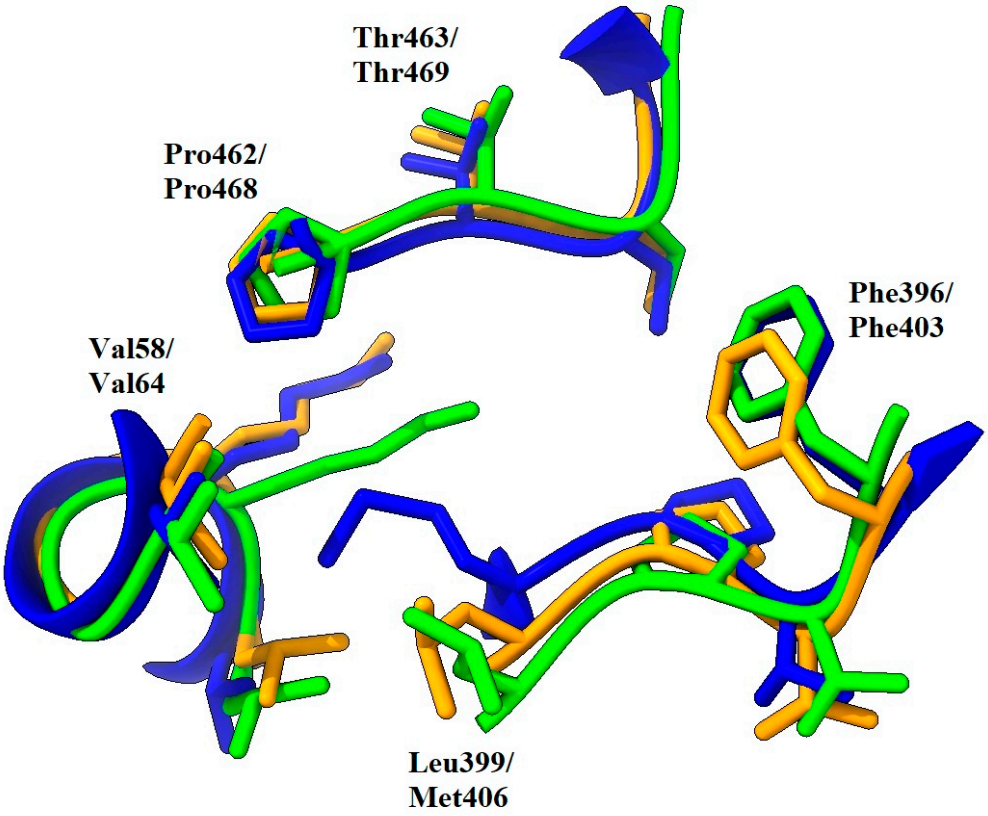

Figure S24. Overlay of TRLMS σ-site with the PDBIDs 5S9W and 3DJJ. TRLMS = green; 5S9W = orange; 3DJJ = blue.

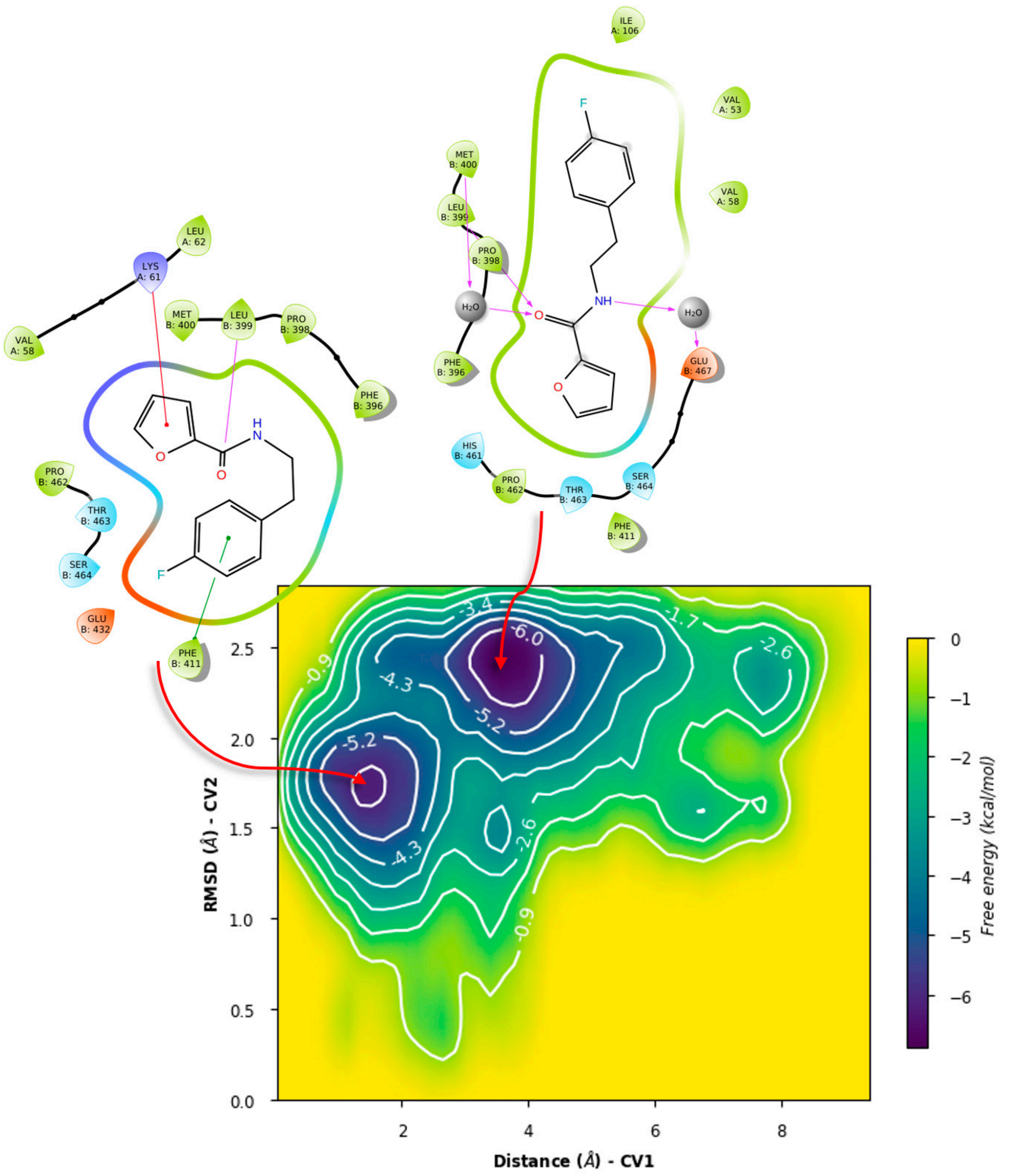

Figure S25. Free energy surface for PLANTS´ top pose of compound 71 in TRLMS (independent replica).

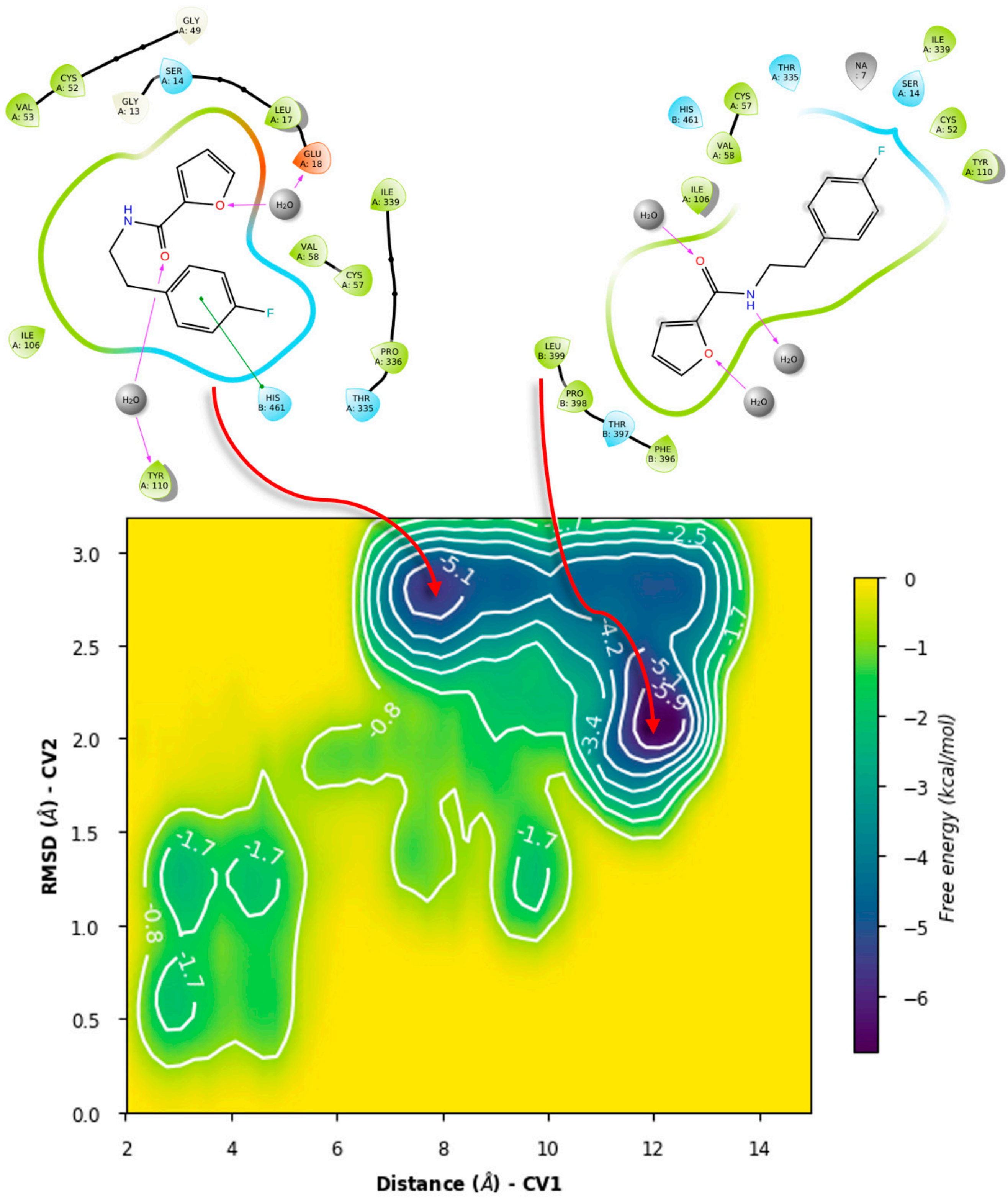

Figure S26. Free energy surface for LeDock top pose of compound 71 in TRLMS (independent replica).

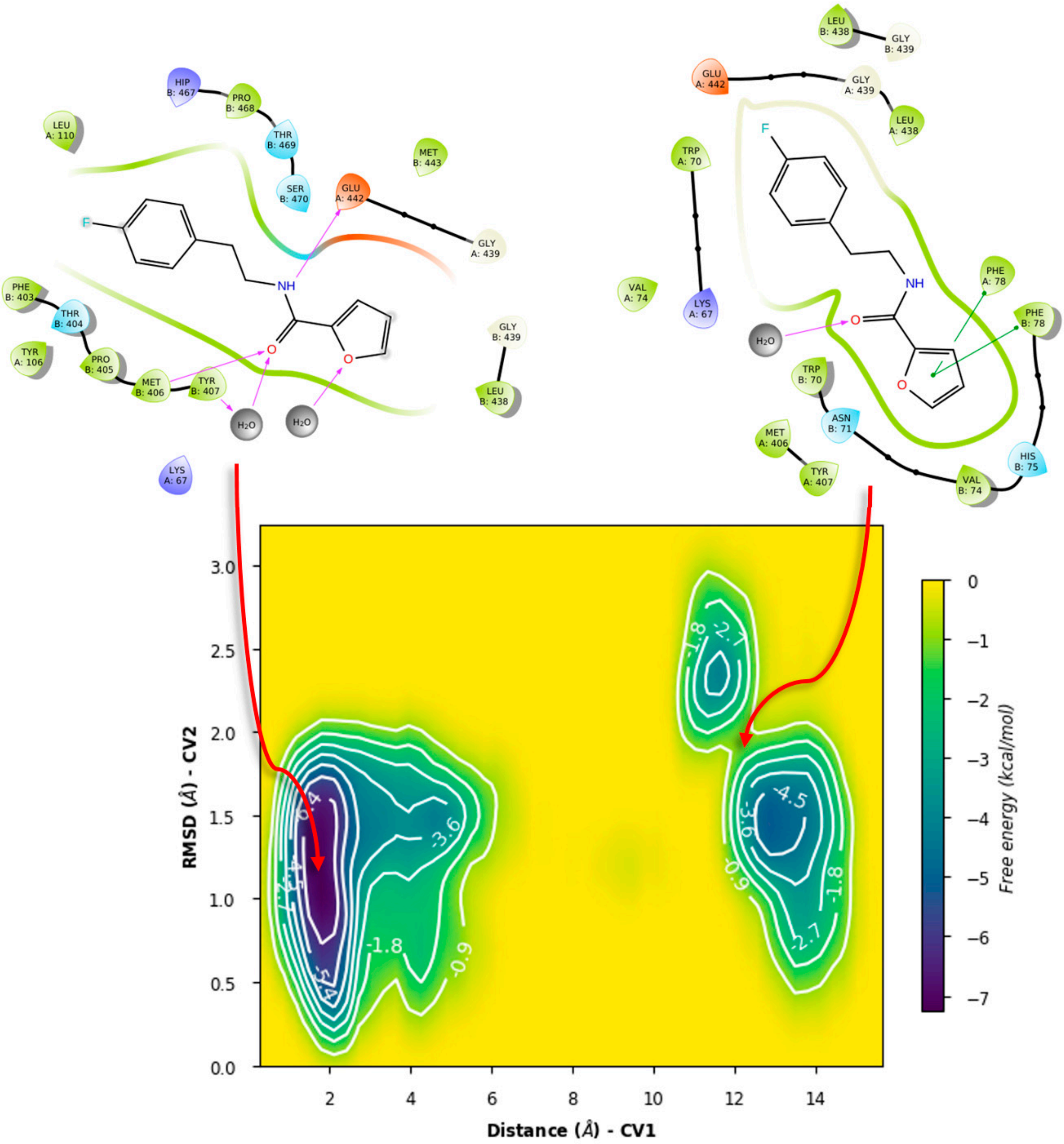

Figure S27. Free energy surface for PLANTS' top pose of compound 71 in GR (independent replica).

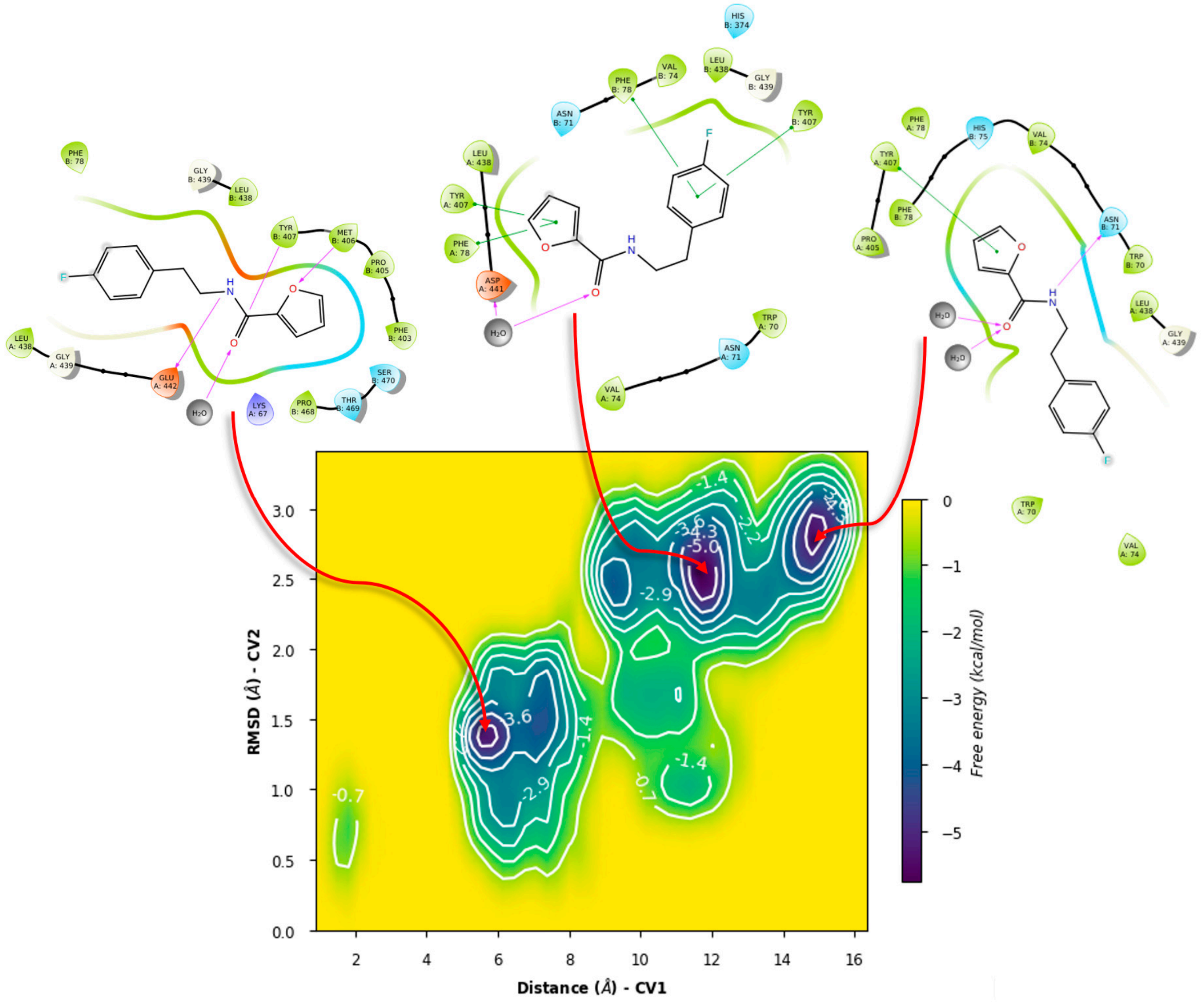

Figure S28. Free energy surface for LeDock's top pose of compound 71 in GR (independent replica).

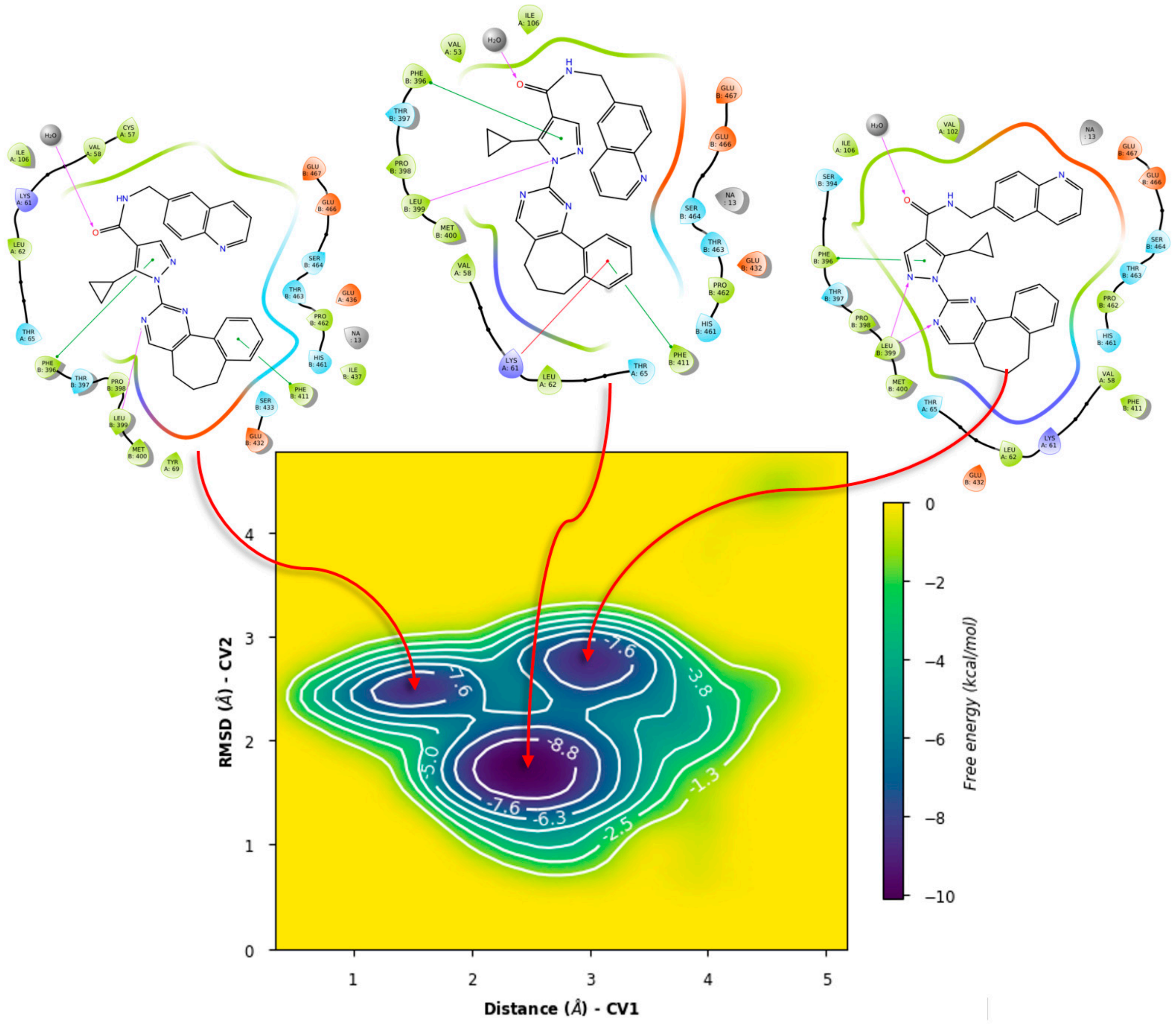

Figure S29. Free energy surface for PLANTS' top pose of ZINC12151998 in TRLMS (independent replica).

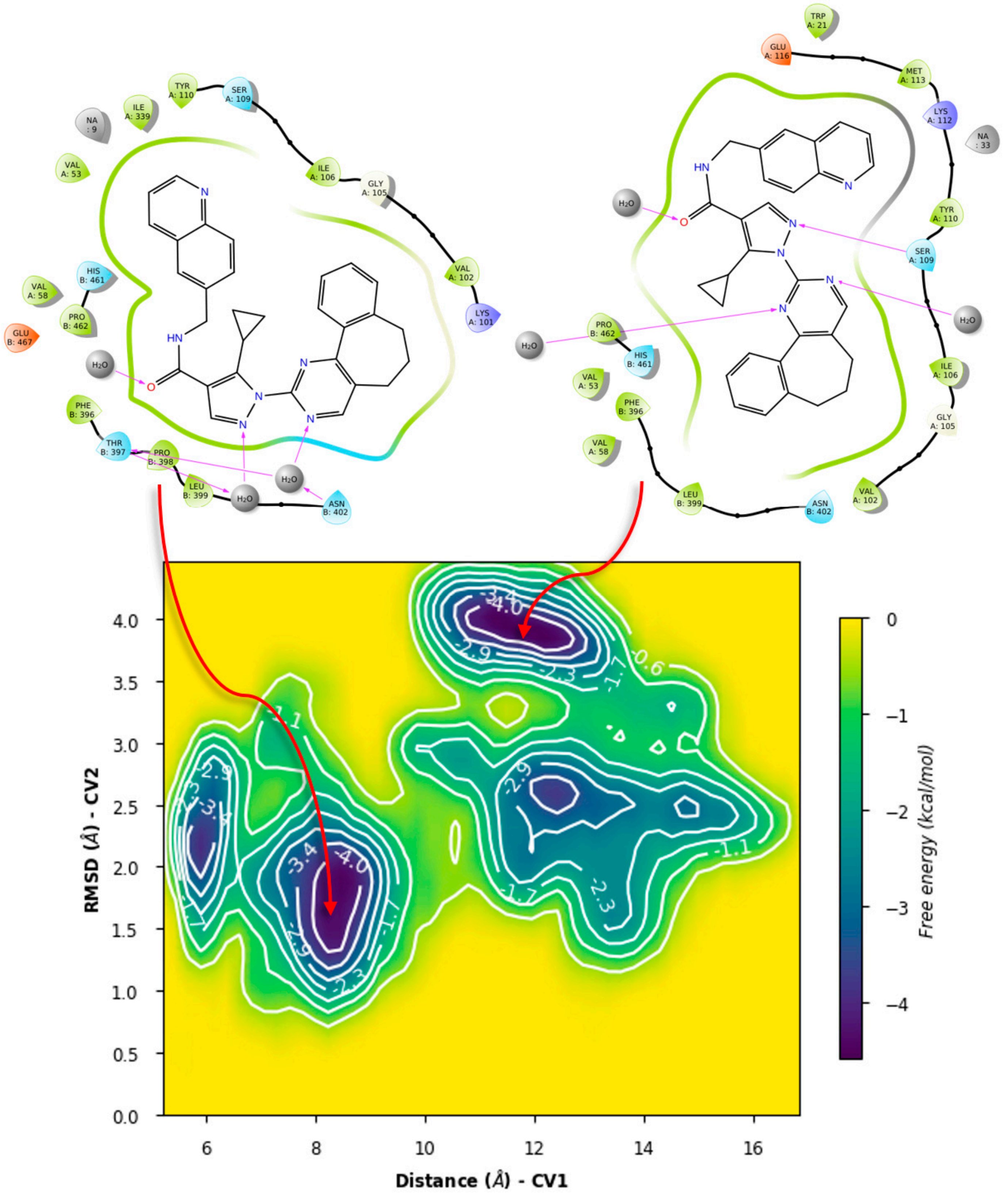

Figure S30. Free energy surface for LeDock's top pose of ZINC12151998 in TRLMS (independent replica).

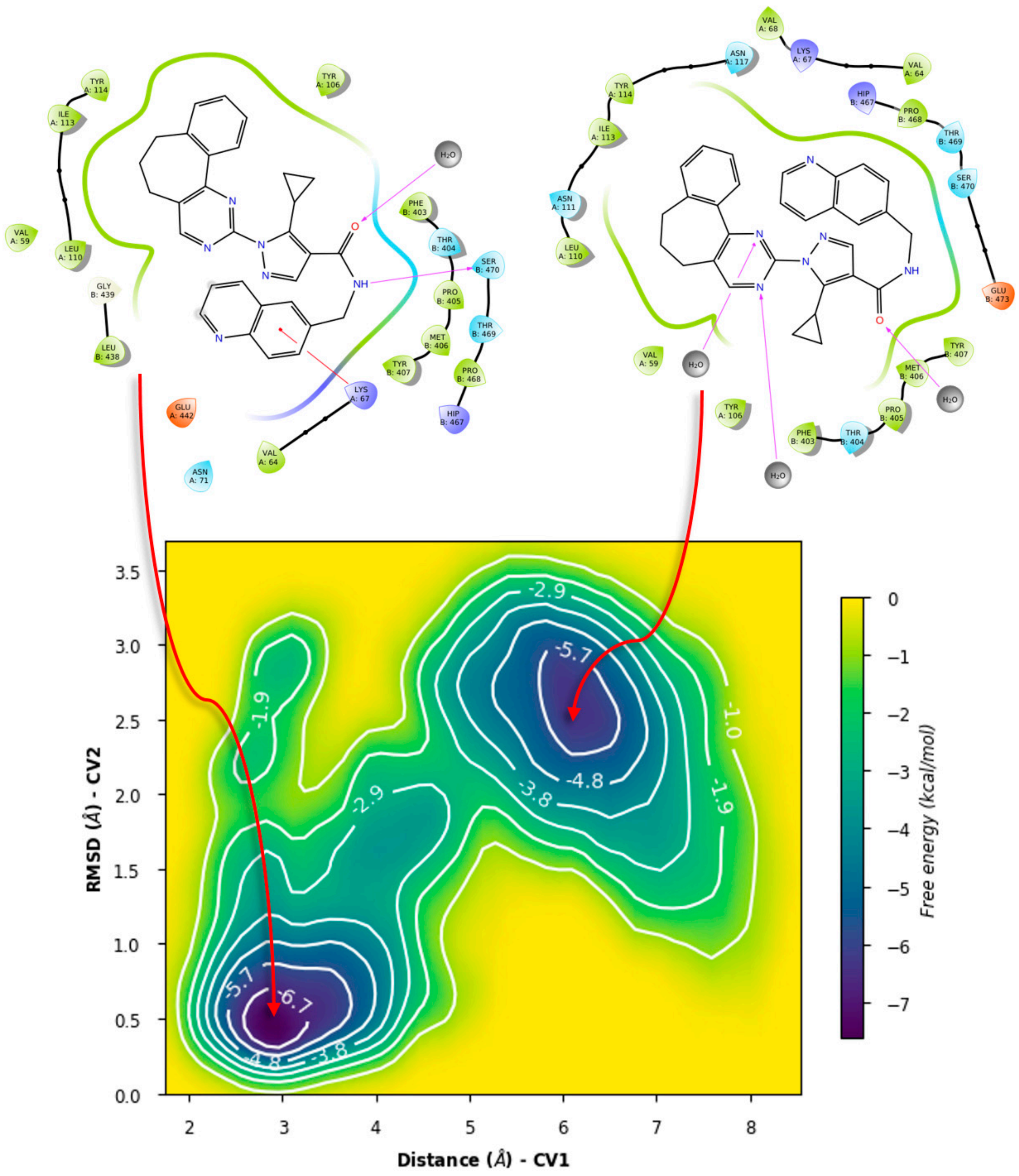

Figure S31. Free energy surface for PLANTS' top pose of ZINC12151998 in GR (independent replica).

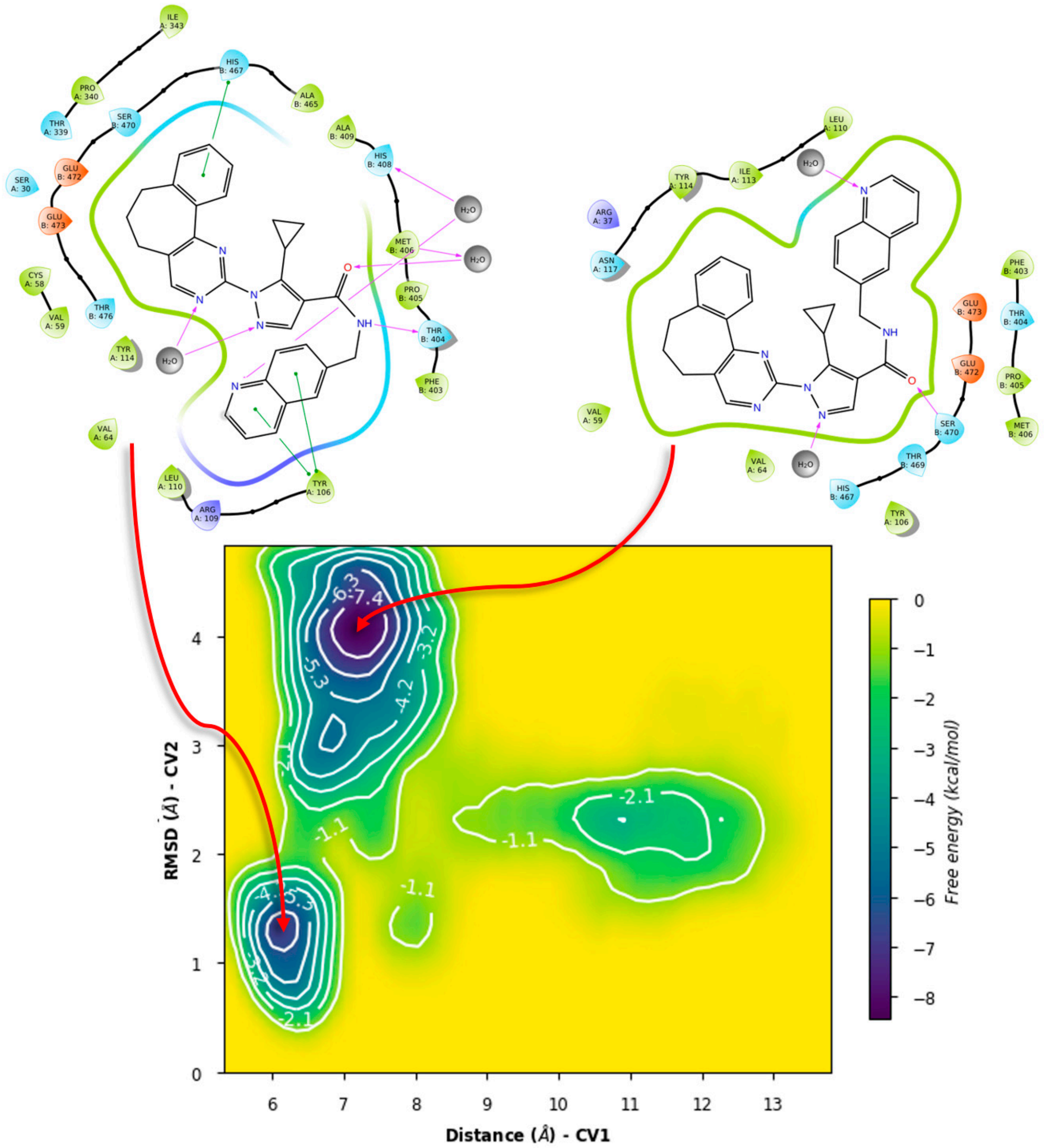

Figure S32. Free energy surface for LeDock's top pose of ZINC12151998 in GR (independent replica).
